# The panda-derived *Lactiplantibacillus plantarum* BSG201683 improves LPS-induced intestinal inflammation and epithelial barrier disruption in vitro

**DOI:** 10.1186/s12866-023-02928-4

**Published:** 2023-09-06

**Authors:** Yi Zhou, Ling Duan, Yan Zeng, Xu Song, Kangcheng Pan, Lili Niu, Yang Pu, Jiakun Li, Abdul Khalique, Jing Fang, Bo Jing, Dong Zeng, Bairong Shen, Xueqin Ni

**Affiliations:** 1 Animal Microecology Research Center, College of Veterinary Medicine, Sichuan Agricultural University, Chengdu, 611130 Sichuan China; 2 Department of Urology and Institutes for Systems Genetics, Frontiers Science Center for Disease-Related Molecular Network, West China Hospital, Sichuan University, Chengdu, 611130 Sichuan China; 3 Animal Feed Affairs of Sichuan Province, Sichuan Provincial Department of Agriculture and Rural Affairs, Chengdu, 610041 Sichuan China; 4 Chengdu Wildlife Institute, Chengdu Zoo, Chengdu, 610081 Sichuan China

**Keywords:** Probiotic, *Lactobacillus*, Inflammation, Permeability, Tight junction

## Abstract

Captive pandas are suffering from intestinal infection due to intestinal microbiota characterized by a high abundance of *Enterobacteriaceae* induced by long-term captivity. Probiotic supplements showed improvement in intestinal barrier function and inflammation. However, the effects of panda-derived probiotics on the intestinal epithelium and inflammation have not been elucidated. In the present study, lipopolysaccharide (LPS) impaired Caco-2 and RAW264.7 inflammatory models were applied to assess the protection of *Lactiplantibacillus plantarum* BSG201683 (*L. plantarum* G83) on barrier disruption and inflammation. The results showed that treatment with *L. plantarum* G83 significantly decreased the paracellular permeability to fluorescein isothiocyanate conjugated dextran (MW 4000, FITC-D4) after LPS induction. Meanwhile, *L. plantarum* G83 alleviated the reduction in tight junction (TJ) proteins and downregulated proinflammatory cytokines caused by LPS in Caco-2 cells. *L. plantarum* G83 also significantly decreased the expression and secretion of pro-inflammatory cytokines in LPS-induced RAW264.7 cells. In addition, the IL-10 increased in both Caco-2 and RAW264.7 cells after *L. plantarum* G83 treatment. The phagocytosis activity of RAW264.7 cells was significantly increased after *L. plantarum* G83 treatment. Toll-like receptor 4/ nuclear factor kappa-B (TLR4/NF-κB) signaling pathways were significantly down-regulated after *L. plantarum* G83 intervention, and the phosphorylation of NF-κB/p65 was consistent with this result. Our findings suggest that *L. plantarum* G83 improves intestinal inflammation and epithelial barrier disruption in vitro.

**Supplementary Information:**

The online version contains supplementary material available at 10.1186/s12866-023-02928-4.

## Background

Dietary intake is an important factor in determining the composition of gut microbiota [1]. For example, studies have shown that Prevotella is increased in abundance in populations consuming a diet rich in plant-based food, while Bacteroides are increased in populations consuming high animal fats and proteins [2]. Consistent with the notion that diet drives the microbial communities, a study using *Cynomolgus macaques* found significant differences in the gut microbiota after 2.5 years of Western- and Mediterranean-type diet consumption [3]. In captive pandas, it has also been shown the environment, and likely the diet, influences the gut microbiota. Pandas in the wild have been shown to have higher levels of *Pseudomonadaceae*. Whereas captive pandas have an expansion of *Enterobacteriaceae* after long-term artificial feeding and antibiotic use [4]. *Enterobacteriaceae* harbors multiple gut pathobionts and expansion of this group has been associated with several disease states [5]. The intestinal barrier is employed as a front line against antigens from the intestinal microbiota and dietary food [6]. As a critical participant in the intestinal barrier, TJ proteins are important to maintain epithelial permeability. There are several proteins involved in response to regulate permeability to prevent microbes and antigens from invading the host via paracellular pathway, including Zonula occludens (ZO), Occludin, Tricellulin, and Claudin [7]. Therefore, impaired intestinal permeability is observed during infection or microbiota dysbiosis. It is demonstrated that the abundance of specific bacteria will increase when microbiota dysbiosis is induced by pathogen infection. The intercellular cross-talk between host and gut microbiota will be subsequently changed [8, 9]. Yao [4] demonstrated that the dominant microbiota in captive pandas is *Enterobacteriaceae*, while that of wild pandas is *Pseudomonadaceae*. Regrettably, most of the reported infection cases in captive pandas are induced by bacteria belonging to *Enterobacteriaceae* [10]. It follows that the integrity of intestinal epithelium will be interrupted and increase the permeability by pathogenic bacteria infection in captive giant pandas, which in turn leads to antigen materials entering the intestinal lamina propria. In addition, the epithelium damage induced by undigested bamboo fibers and parasitic infections in giant pandas will also cause intestinal inflammation. Intestinal microbiota plays an important role in maintaining intestinal barrier function.

Probiotics, as a diary supplement, act on host health in multiple possible ways including intestinal epithelial barrier maintenance and immune modulation [11]. It has been speculated that *Lactobacillus* species beneficially modulate the expression of tight junction protein, thereby promoting barrier integrity. Many studies suggested that *L. casei* [12], *L. plantarum* [13], or *L. rhamnosus* [14] caused a modest enhancement in the intestinal epithelial TJ proteins. There are many inflammatory pathways involved during the pathogen infection, and it can be triggered by *Lactobacillus* spp. to regulate intestinal permeability, helping TJ proteins assemble and redistribute on the cell surface [15].

In addition, the *Lactobacillus* spp. is known as an active immune mediator through interacting with many antigen-presenting cells (APCs) [16]. There are many pathogen-associated molecular patterns (PAMPs) in intestinal content that regulate intestinal immunity by interacting with pattern recognition receptors (PRRs), such as Toll-like receptors [17, 18]. Many studies demonstrated that the *Lactobacillus* spp. regulates the immune response by triggering off the TLR2 relative pathways to boost or inhibit pro-inflammatory cytokine release [18–20]. Although *Lactobacillus* can’t directly activate TLR4, however, it can indirectly participate in the regulation of TLR4 by affecting the intestinal microbiota [21]. *Lactobacillus* spp. can recruit myeloid differentiation factor 88 (MyD88) through TLR signal transduction and the phosphorylated IRAK will interact with TRAF6 to transmit downstream signals, which in turn induces inflammatory cytokines production (Interleukin-1β (IL-1β), IL-6, IL-8, IL-12, INF-γ, and TNF-α) and chemokines [22, 23].

In our previous study, we demonstrated that *L. plantarum* G83 isolated from the giant panda feces showed an effective probiotic profile in *vitro* and in *vivo* studies. However, the regulation of epithelial barrier function and inflammatory response by *L. plantarum* G83 is still unexplored. To address this concern, the LPS-impaired Caco-2 and RAW264.7 inflammatory models were applied to assess the protection of *L. plantarum* G83 on LPS-induced barrier disruption and inflammation.

## Results

### Effects of *L. plantarum* G83 on cell viability

**Fig. 1:**
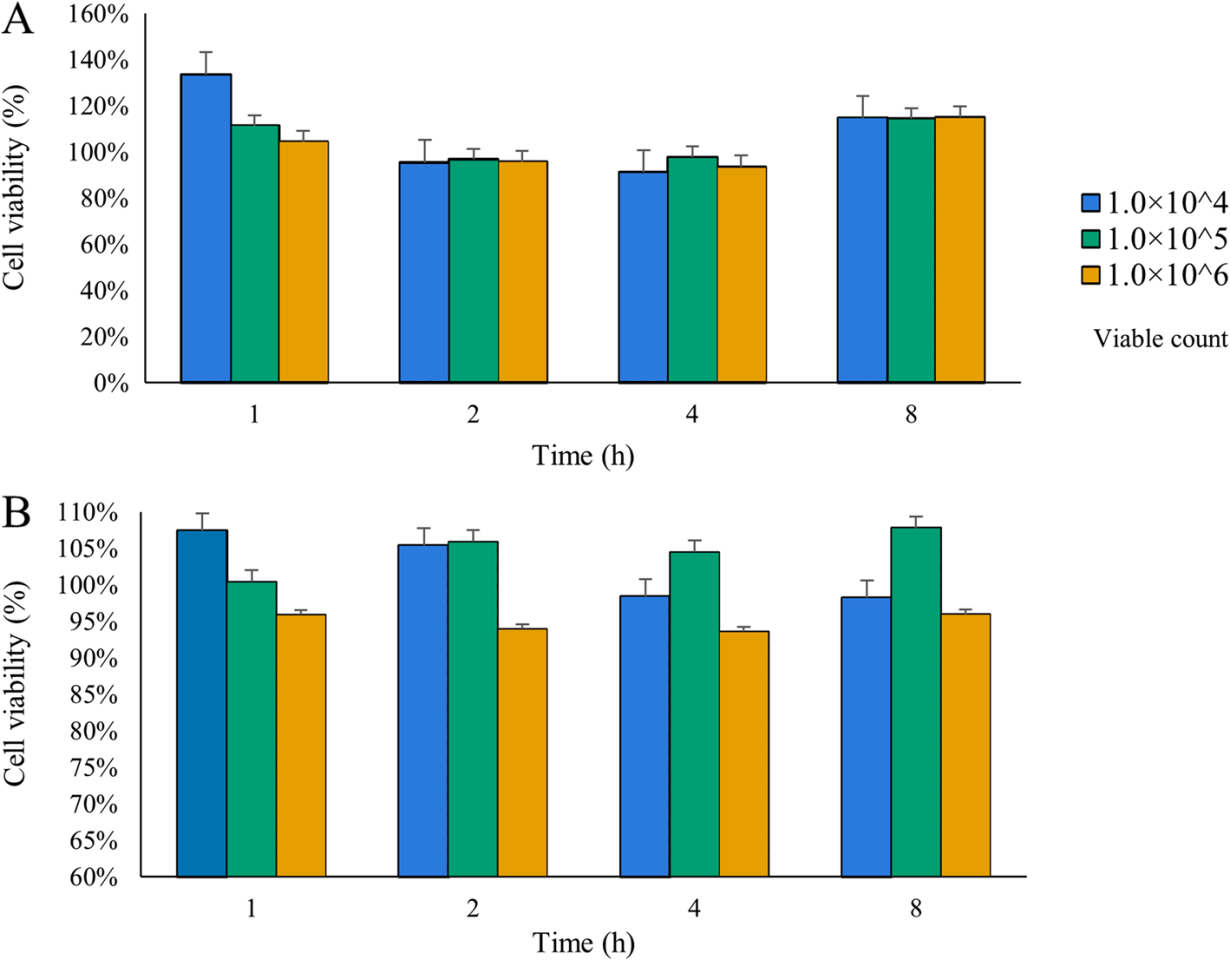
Effects of different concentrations of *L. plantarum* G83 on the viability of Caco-2 (**A**) and RAW264.7 (**B**) cells in different treatment time

RAW264.7 and Caco-2 cells were co-cultured with different viable counts of *L. plantarum* G83 at 1:1 (1.0 × 10^4^ CFU), 1:10 (1.0 × 10^5^ CFU) and 1:100 (1.0 × 10^6^ CFU) for 1, 2, 4 and 8 h, respectively. The results showed that no significant cell damage was observed under microscope, and the cell viability ratio was greater than 95% in all treatment groups. It is indicated that *L. plantarum* G83 had no significant cytotoxicity to Raw264.7 or Caco-2 cells (Fig. 1).

### Validation of Caco-2 cell monolayer

**Fig. 2:**
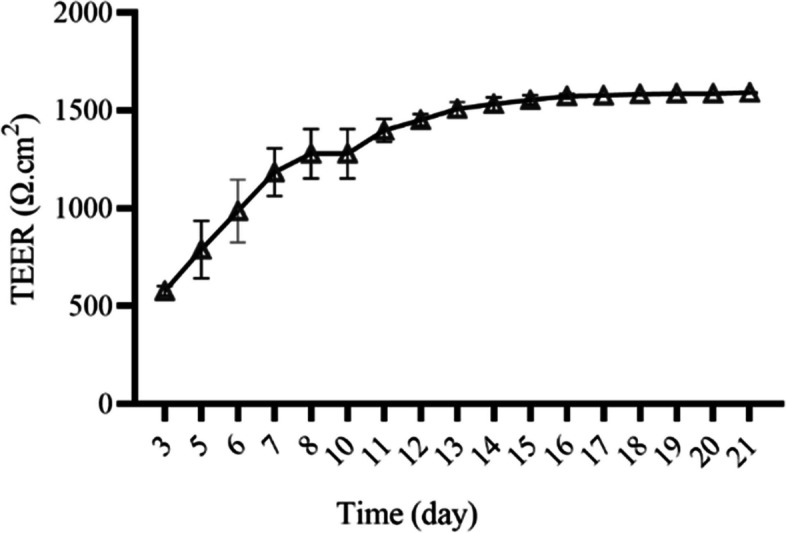
Transepithelial electrical resistance (TEER) changes over time

As shown in Fig. 2, TEER increased rapidly from day 3 to day 7, and it slowed down in the following two weeks. The TEER had very little change from day 17 to day 21. Thus, the Caco-2 cell monolayer was successfully formed on day 21.

### *L. plantarum* G83 decreases paracellular permeability in LPS-impaired Caco-2 cell

**Fig. 3:**
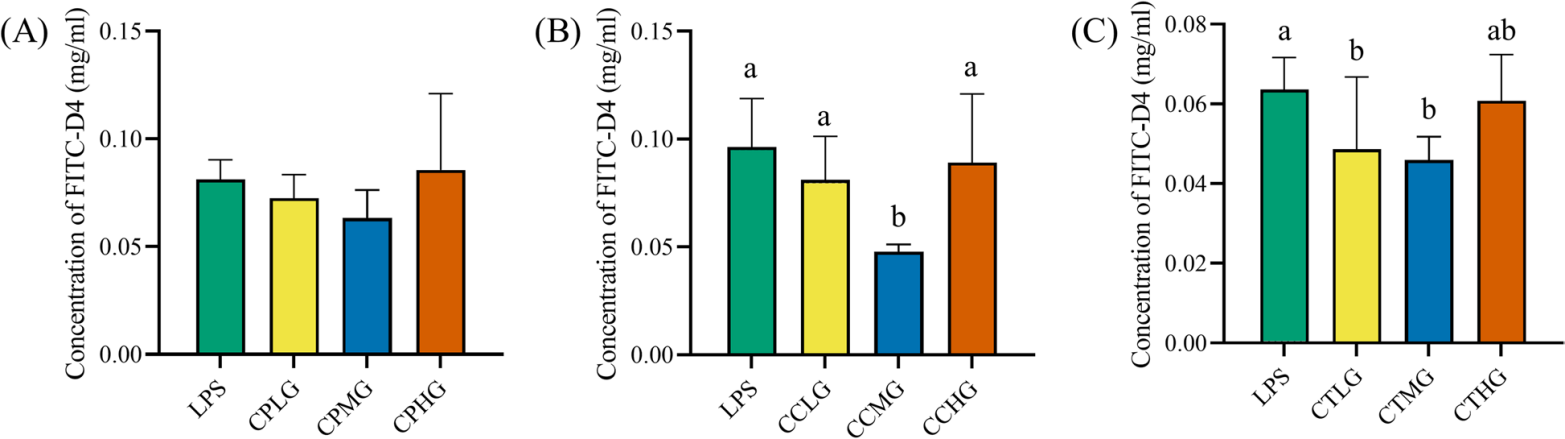
Effects of different treatments of *L. plantarum* G83 on permeability LPS-induced Caco-2 cell monolayer. The permeability of Caco-2 cell monolayer was determined by transmittance of FITC-D4. Results of pre-treatment groups (**A**), co-culture groups (**B**) and post-treatment groups (**C**) are shown as mean ± SD. Data are analyzed with one-way ANOVA (Tukey's test). Different letters mean *p* < 0.05, same letter means *p* > 0.05

Caco-2 cell monolayer was used to investigate the effect of *L. plantarum* G83 on intercellular transport. The monolayer cell model was successfully generated on the transwell system on day 21. There was a lower FITC-D4 fluorescence intensity observed in the CCMG group compared with low and high co-culture group. Likewise, CTLG and CTMG groups showed a lower fluorescence intensity compared with other groups among the post-treatment groups. However, we didn’t see any difference among the pre-treatment groups (Fig. 3). It indicated that appropriate counts of *L. plantarum* was associated with enhanced permeability in Caco-2 cell monolayer.

### *L. plantarum* G83 promotes intercellular junctions in LPS-impaired Caco-2 cell

**Fig. 4:**
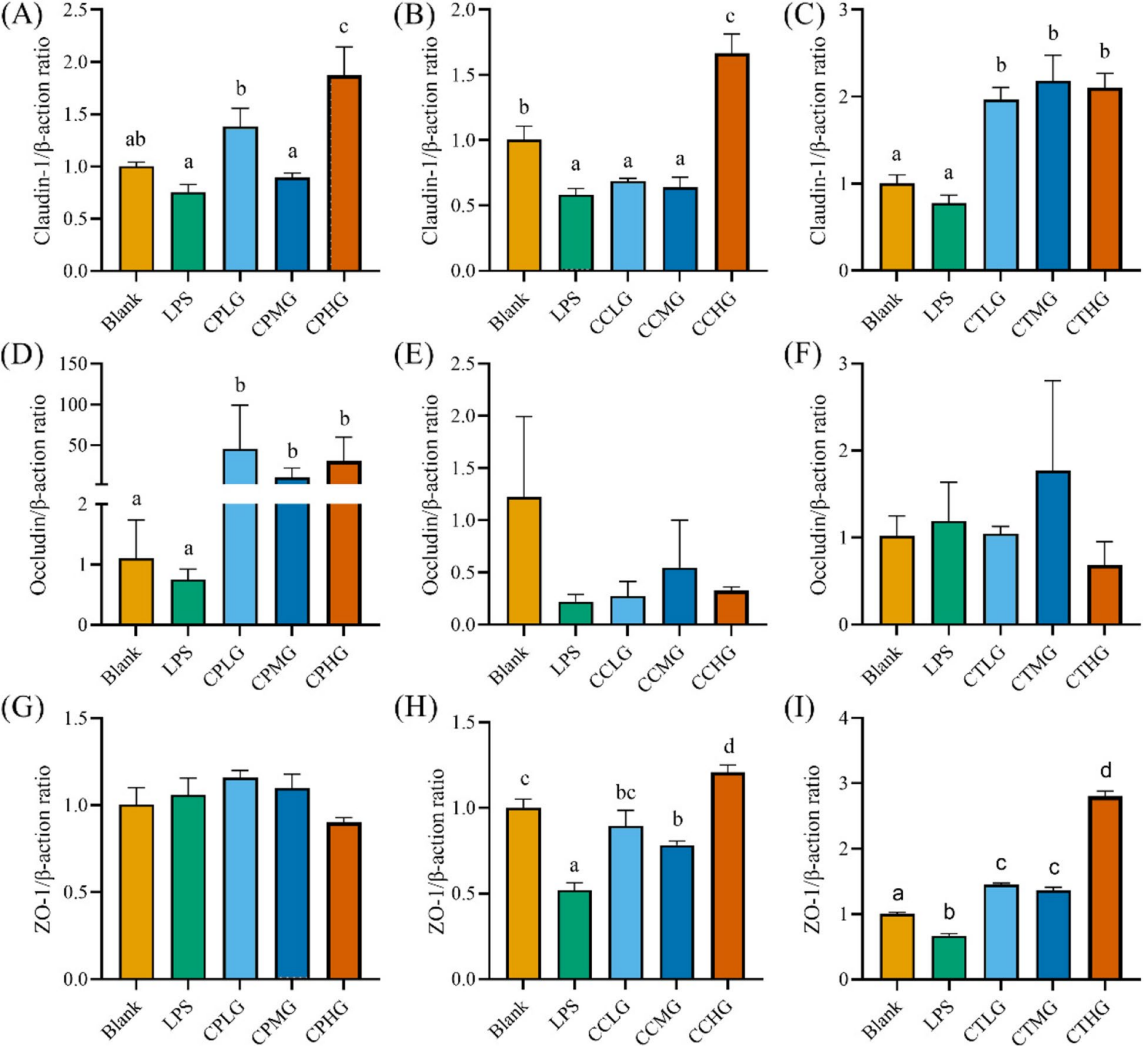
Effects of different treatments of *L. plantarum* G83 on tight junction protein mRNA expression in LPS-impaired Caco-2 cells. Claudin-1 (**A-C**), occludin (**D-F**) and ZO-1 (G-I) gene expression levels were determined by qRT-PCR after Caco-2 pre-treated (**A**, **D**, **G**), co-cultured (**B**, **E**, **H**) and post-treated (**C**, **F**, **I**) with different viable count of *L. plantarum*. All data are shown as mean ± SD. Data are analyzed with one-way ANOVA (Tukey's test). Different letters mean *p* < 0.05, same letter means *p* > 0.05

**Fig. 5:**
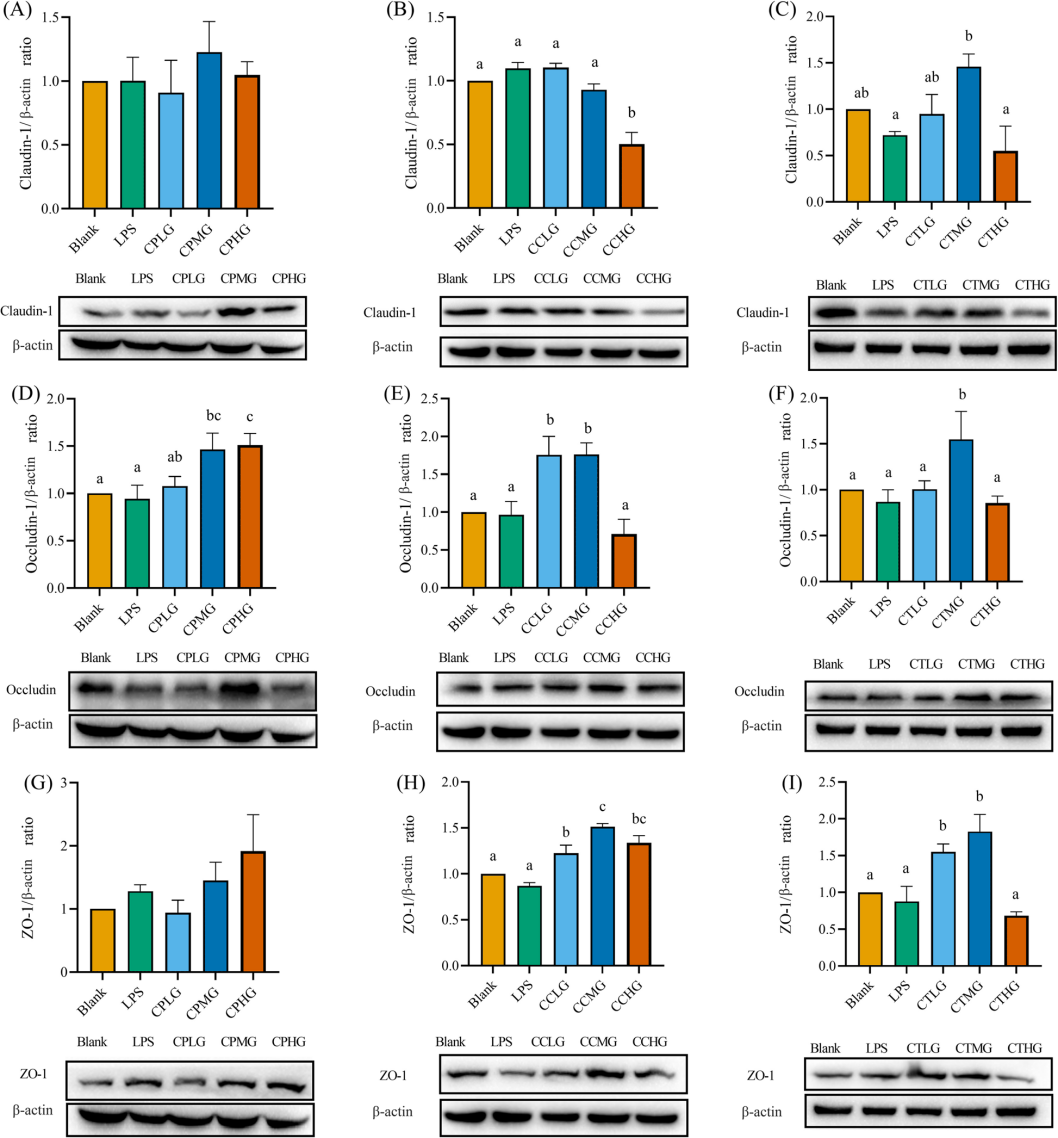
Effects of different treatments of *L. plantarum* G83 on Tight junction protein mRNA expression in LPS-impaired Caco-2 cell. Claudin-1 (**A**-**C**), occludin-1 (**D**-**F**) and ZO-1 (**G**-**I**) protein expression level were determined by western blot after Caco-2 pre-treated (**A**, **D**, **G**), co-cultured (**B**, **E**, **H**) and post-treated (**C**, **F**, **I**) with different viable count of *L. plantarum* G83. The same batch of different treatment samples ran on the same gel and blots closest to the mean abundance cropped from different gels were presented in the figure. All data are shown as mean ± SD. Data are analyzed with one-way ANOVA (Tukey's test). Different letters mean *p* < 0.05, same letter means *p* > 0.05

To confirm the beneficial effect of *L. plantarum* G83 on TJ proteins of Caco-2 cell, RT-qPCR and western blot were performed to analyze the Claudin-1, Occludin, and ZO-1 after different viable counts of *L. plantarum* G83 treatment. The Claudin-1 mRNA was up-regulated in the CPLG and CPMG groups. Furthermore, only the CCHG group showed a higher expression level of Claudin-1 gene in co-culture treatment groups. However, all three treatment groups showed a capability to improve the Claudin-1 gene expression compared with that in the LPS treatment group (Fig. 4A-C). The Occludin mRNA was highly improved in all pre-treatment groups, however, there was no significant difference observed in co-culture and post-treatment groups (Fig. 4D-F). Besides, CPLG and CPHG groups had a light promotion effect on the ZO-1 gene expression. In addition, co-culture and post-treatment groups can increase the ZO-1 mRNA expression level (Fig. 4G-I). The western blot results of TJ proteins were consistent with the gene expression result. As shown in Fig. 5, there was no difference in the protein level of Claudin-1 in the pre-treatment group. No difference was found in the CCLG and CCMG groups when compared with that of the LPS group. Whereas the high dose of *L. plantarum* G83 significantly reduced the expression of Claudin-1 protein. In the post-treatment groups, low-and medium-dose treatment groups increased the Claudin-1 protein expression, but the high-dose group showed the opposite result. Meanwhile, the *L. plantarum* G83 showed a tendency to promote ZO-1 protein expression in the pre-treatment and co-culture groups, but there was no significant difference. Furthermore, CTMG and CTHG groups showed a significant effect on enhancing ZO-1 protein expression. Besides, the expression of Occludin protein in the CPLG and CPMG groups was significantly increased, while that of the CPHG group was slightly decreased. Meanwhile, the Occludin protein only showed an increasing trend in medium dose treatments of co-culture and post-treatment group, other treatments had no difference compared with the LPS group (Fig. 5).

### *L. plantarum* G83 reduces inflammatory cytokine release in LPS-induced RAW264.7 and Caco-2 cell

**Fig. 6:**
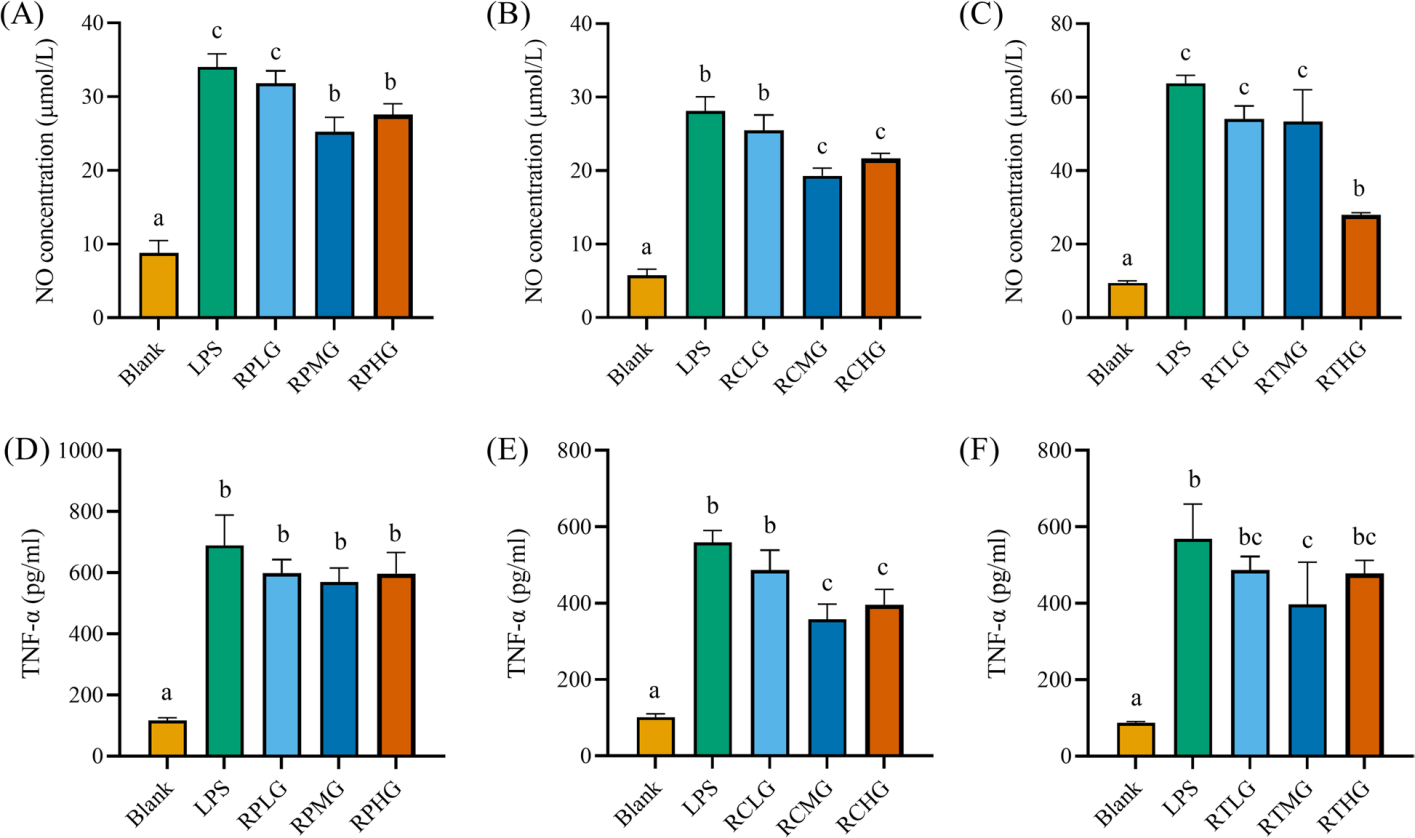
Effects of different treatments of *L. plantarum* G83 on the secretion of NO and TNF-α in LPS-induced RAW264.7 cell. NO (**A**-**C**) and TNF-α (**D**-**F**) in the RAW264.7 cell culture supernatant were determined by ELISA after RAW264.7 pre-treated (**A** and **D**), co-cultured (**B** and **E**) and post-treated (**C** and **F**) with different viable count of *L. plantarum* G83. All data are shown as mean ± SD. Data are analyzed with one-way ANOVA (Tukey's test). Different letters mean *p* < 0.05, same letter means *p* > 0.05

**Fig. 7:**
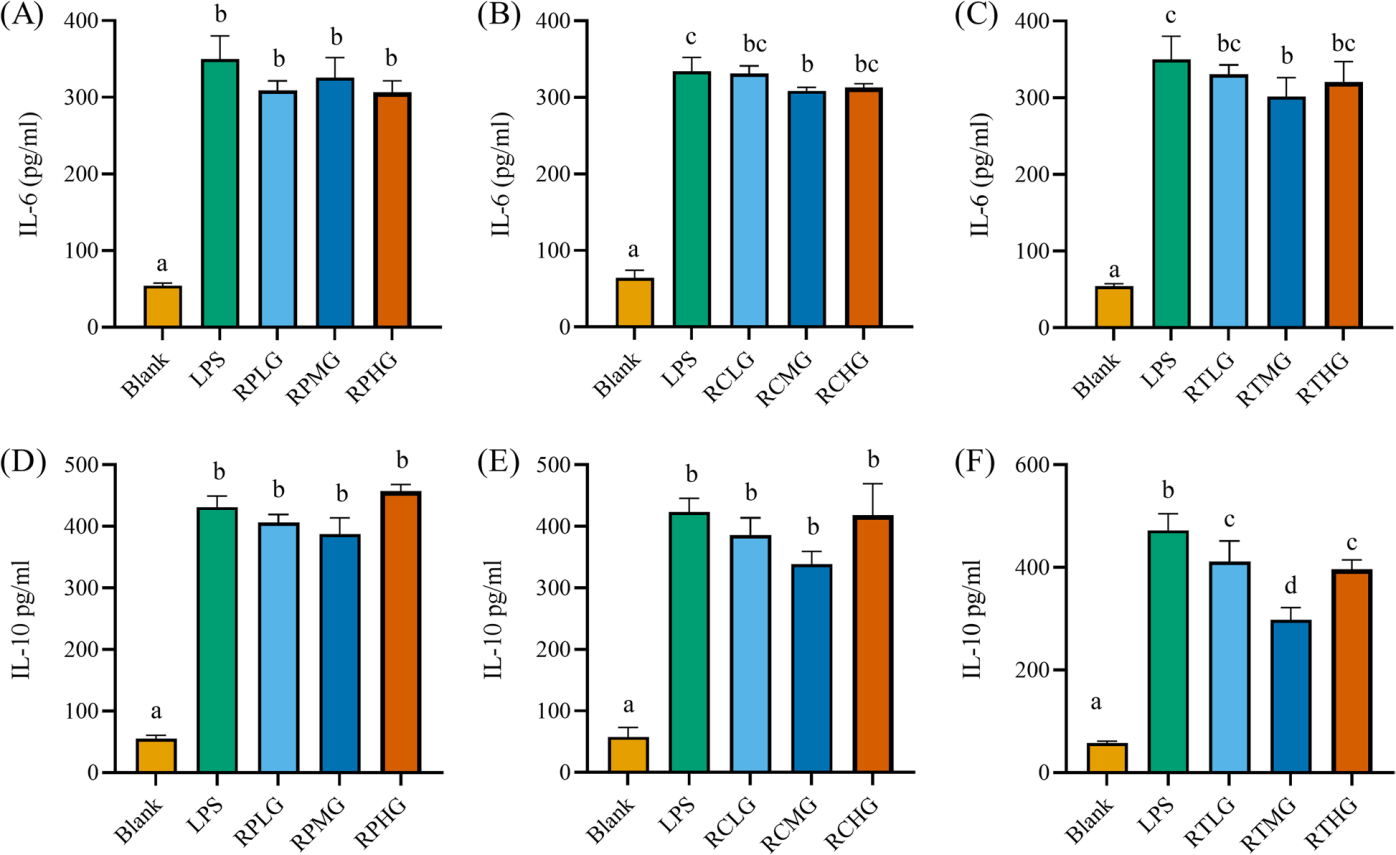
Effects of different treatments of *L. plantarum* G83 on the secretion of IL-6 and IL-10 in LPS-induced RAW264.7 cell. IL-6 (**A**-**C**) and IL-10 (**D**-**F**) in the cell culture supernatant were determined by ELISA after RAW264.7 pre-treated (**A** and **D**), co-cultured (**B** and **E**) and post-treated (**C** and **F**) with different viable count of *L. plantarum* G83. All data are shown as mean ± SD. Data are analyzed with one-way ANOVA (Tukey's test). Different letters mean *p* < 0.05, same letter means *p* > 0.05

To explore whether the *L. plantarum* G83 could alleviate inflammatory response by LPS in RAW264.7 cells, ELISA was performed to determine inflammatory cytokines in the cell culture supernatant. Results showed that massive inflammatory cytokines were released into the supernatant after LPS stimulation. RPMG and RPHG treatment groups showed a capability to inhibit LPS-induced NO release. Also, *L. plantarum* G83 pre-treated groups showed a tendency to reduce the TNF-α, IL-6, and IL-10 in the supernatant (Figs. 6 and 7). Meanwhile, RCMG and RCHG co-culture treatment groups effectively inhibited the secretion of NO and TNF-α (Fig. 6), however, IL-6 was only decreased in the RCMG group. We also get the same decreased tendency of the TNF-α and IL-6 in the RTMG and RTHG groups, but NO concentration was only decreased in the RTHG group. As an anti-inflammatory cytokine, IL-10 significantly increased after LPS stimulation, which may be the result of negative feedback regulation of inflammatory response (Fig. 7).

**Fig. 8:**
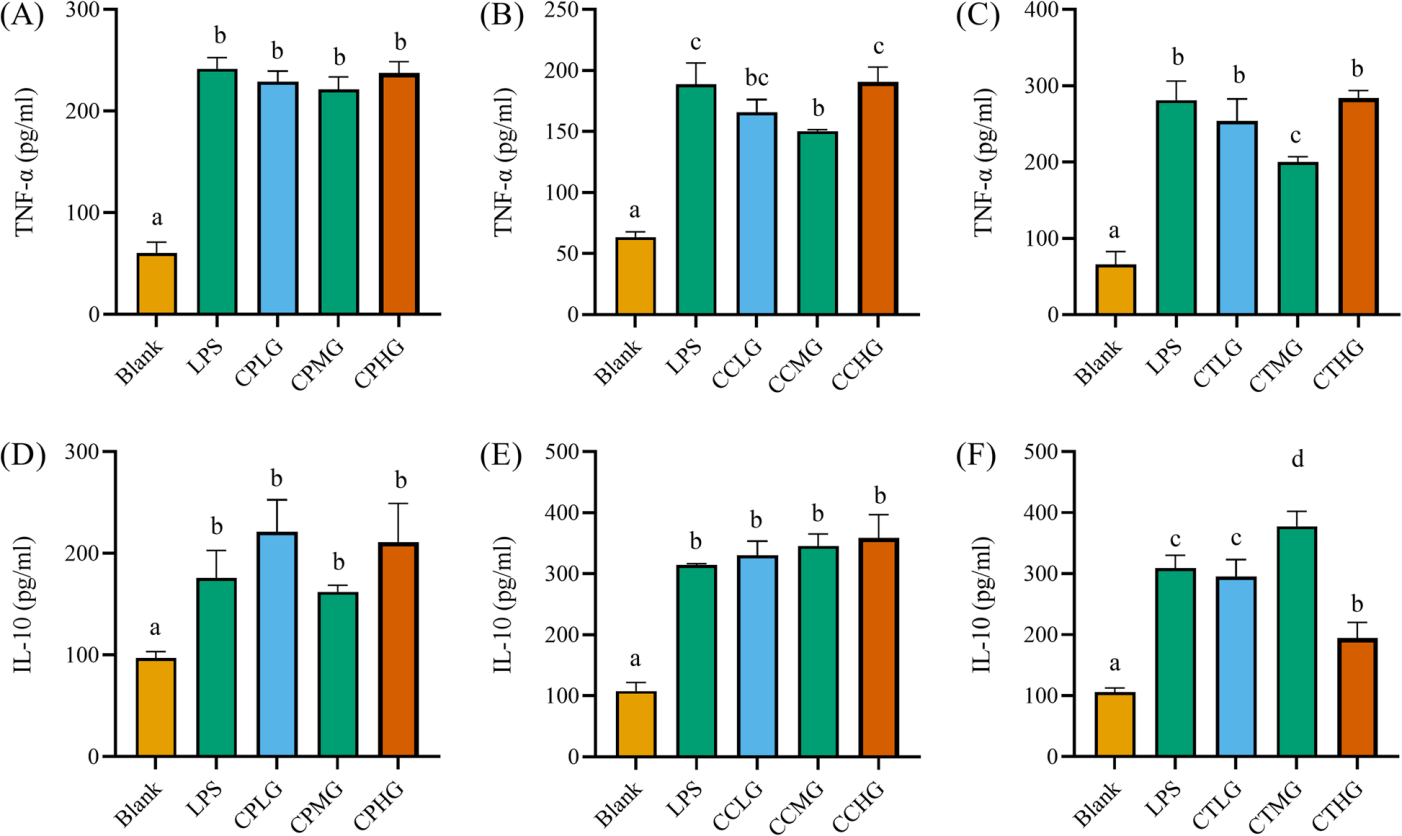
Effects of different treatments of *L. plantarum* G83 on TNF-α and IL-10 in the supernatant of LPS-impaired Caco-2 cells. TNF-α (**A**-**C**) and IL-10 (**D**-**F**) in the cell culture supernatant were determined by ELISA after Caco-2 pre-treated (**A** and **D**), co-cultured (**B** and **E**) and post-treated (**C** and **F**) with different viable count of *L. plantarum* G83. All data are shown as mean ± SD. Data are analyzed with one-way ANOVA (Tukey's test). Different letters mean *p* < 0.05, same letter means *p* > 0.05

We are wondering whether the inflammatory response was improved by *L. plantarum* G83 in LPS-impaired Caco-2 cells. As shown in the figure, the TNF-α was significantly decreased in the supernatant of the CCLG, CCMG, and CTMG treatment groups, while other treatment groups had no difference compared with the LPS group (Fig. 8). It indicated that the *L. plantarum* G83 showed a capability to reduce the inflammatory response in the co-culture and post-treated group. Meanwhile, the concentration of IL-10 in the CTMG group was significantly improved (Fig. 8).

### *L. plantarum* G83 improves the phagocytosis function of RAW264.7

**Fig. 9:**
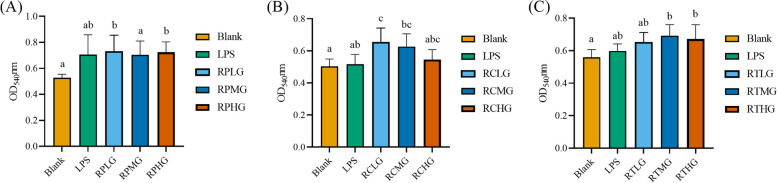
Effects of different treatments of *L. plantarum* G83 on the phagocytic activity of RAW264.7. The phagocytic activity of RAW264.7 were determined by 0.05% neutral red staining after pre-treated (**A**), co-cultured (**B**) and post-treated (**C**) with different viable count of *L. plantarum* G83. All data are shown as mean ± SD. Data are analyzed with one-way ANOVA (Tukey's test). Different letters mean *p* < 0.05, same letter means *p* > 0.05

The phagocytosis activity of RAW264.7 cells was significantly increased in low- and high-dose pre-treatment groups compared with that of the Blank group (*p* < 0.05) (Fig. 9). In comparison, medium-dose and high-dose co-culture groups showed higher phagocytic activity compared to the Blank group (*p* < 0.05). The same result was observed in the medium-dose and high-dose post-treatment groups (*p* < 0.05), but there was no difference compared with that of the LPS group (*p* > 0.05). *L. plantarum* G83 showed promoting effect on the phagocytosis activity of macrophages.

### *L. plantarum* G83 reduces the inflammatory response in LPS-induced RAW264.7 by TLR4/NF-κB pathway

**Fig. 10:**
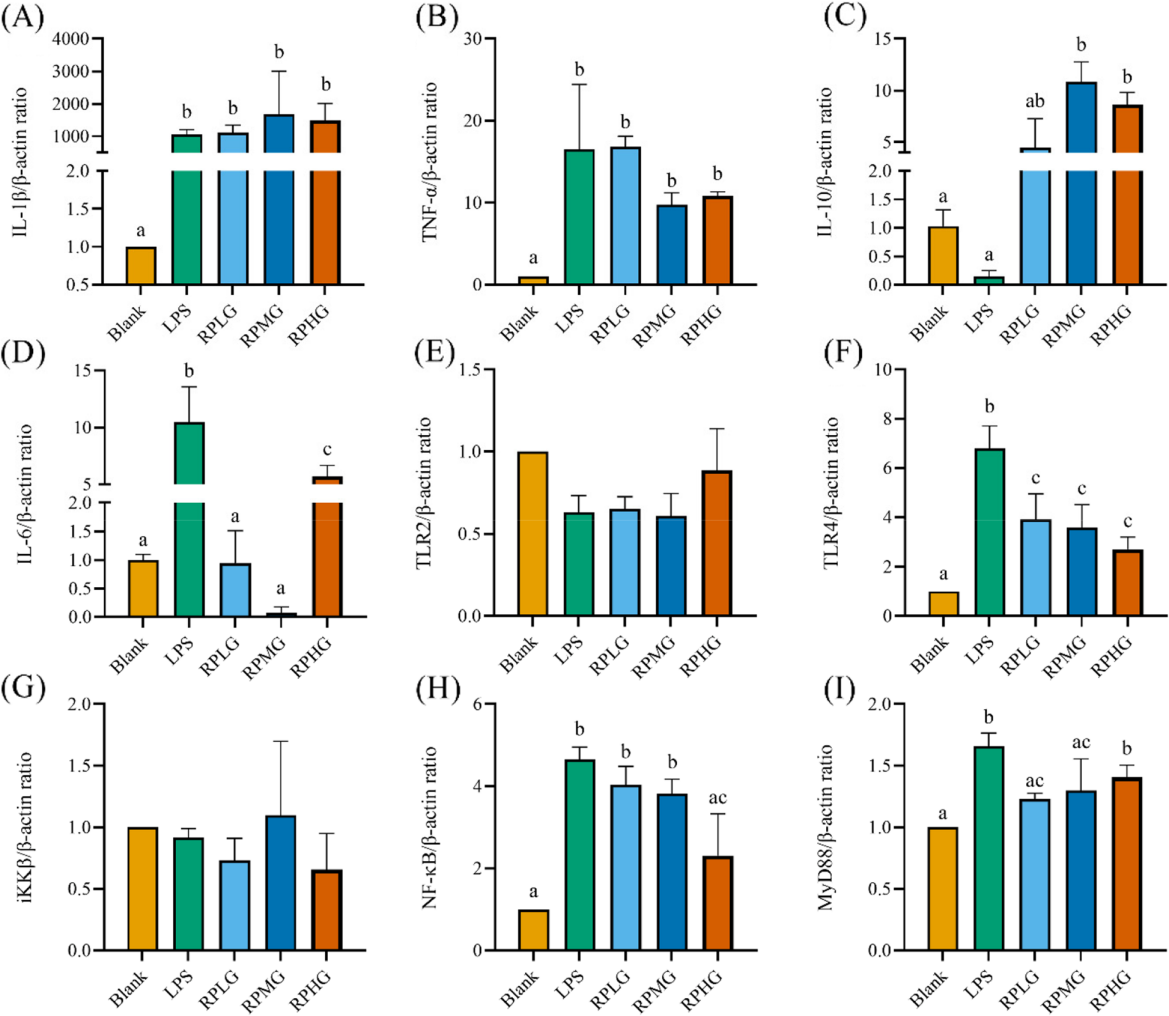
Effects of different doses of *L. plantarum* G83 pre-treated with LPS-induced RAW264.7 on the inflammation relative gene expression. Inflammation related cytokines genes (IL-1β (**A**), TNF-α (**B**), IL-10 (**C**), IL-6 (**D**))and TLRs/NF-κB related genes (TLR2 (**E**), TLR4 (**F**), iKK β (**G**), NF-κB (**H**) and MyD88 (**I**)) were determined by qRT-PCR after RAW264.7 pre-treated with different viable count of *L. plantarum* G83. All data are shown as mean ± SD. Data are analyzed with one-way ANOVA (Tukey's test). Different letters mean *p* < 0.05, same letter means *p* > 0.05

**Fig. 11:**
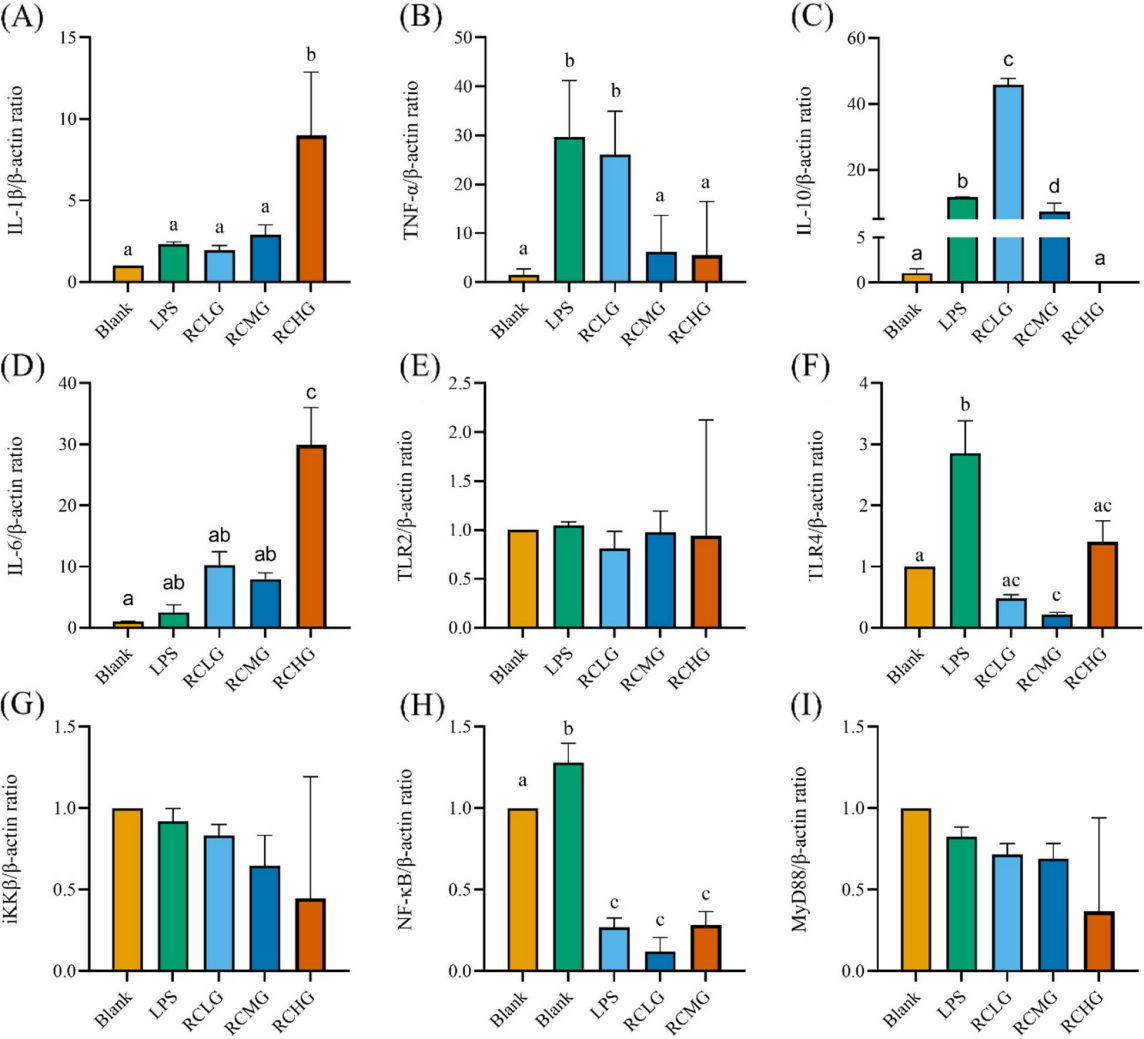
Effects of different doses of *L. plantarum* G83 co-cultured with LPS-induced RAW264.7 on the inflammation relative gene expression. Inflammation related cytokines genes (IL-1β (**A**), TNF-α (B), IL-10 (**C**), IL-6 (**D**))and TLRs/NF-κB related genes (TLR2 (**E**), TLR4 (**F**), iKK β (**G**), NF-κB (**H**) and MyD88 (**I**)) were determined by qRT-PCR after RAW264.7 co-cultured with different viable count of *L. plantarum* G83. All data are shown as mean ± SD. Data are analyzed with one-way ANOVA (Tukey's test). Different letters mean *p* < 0.05, same letter means *p* > 0.05

**Fig. 12:**
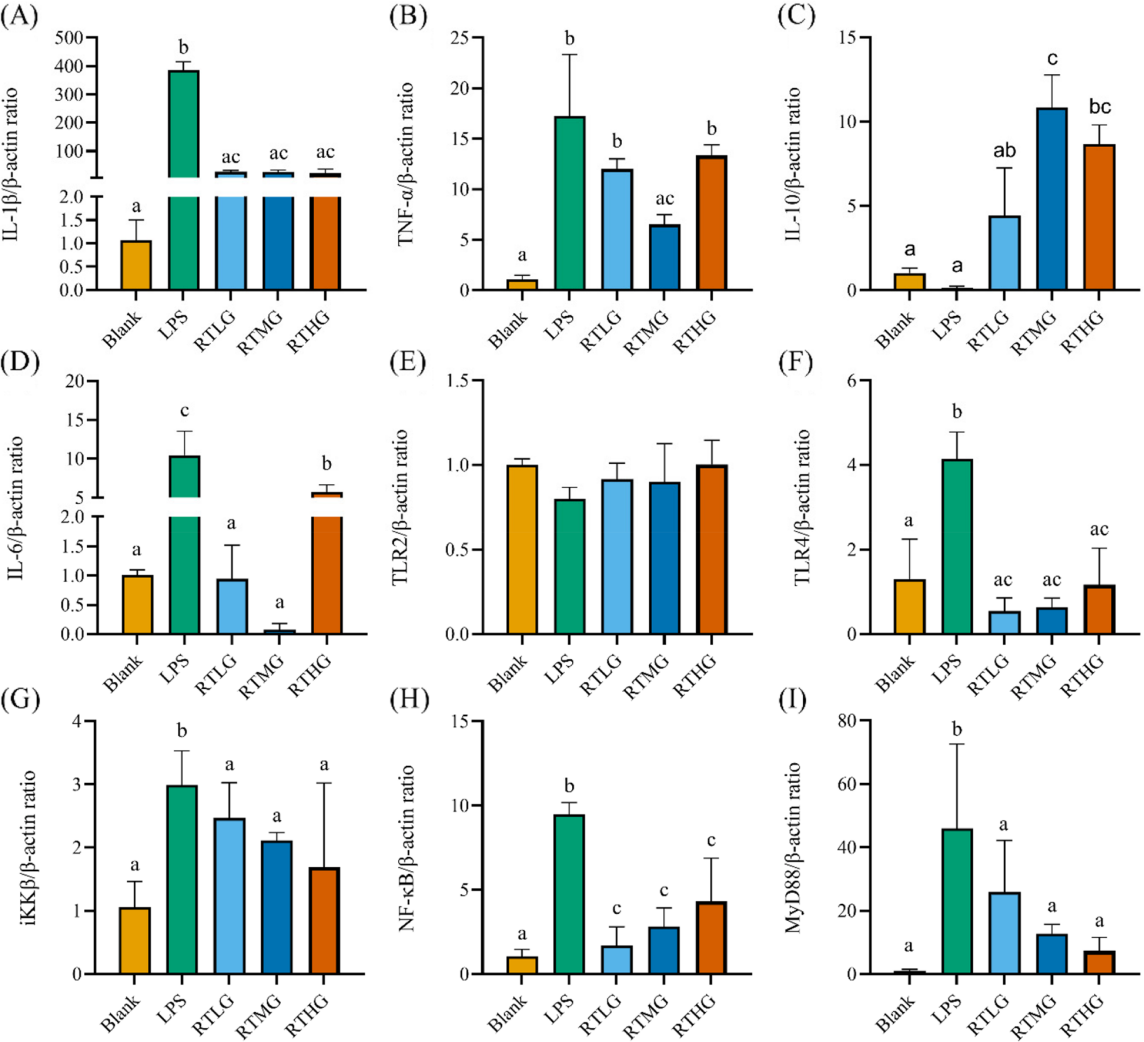
Effects of different doses of *L. plantarum* G83 post-treated with LPS-induced RAW264.7 on the inflammation relative gene expression. Inflammation related cytokines genes (IL-1β (**A**), TNF-α (**B**), IL-10 (**C**), IL-6 (**D**)) and TLRs/NF-κB related genes (TLR2 (**E**), TLR4 (**F**), iKK β (**G**), NF-κB (**H**) and MyD88 (**I**)) were determined by qRT-PCR after RAW264.7 post-treated with different viable count of *L. plantarum* G83. All data are shown as mean ± SD. Data are analyzed with one-way ANOVA (Tukey's test). Different letters mean *p* < 0.05, same letter means *p* > 0.05

**Fig. 13:**
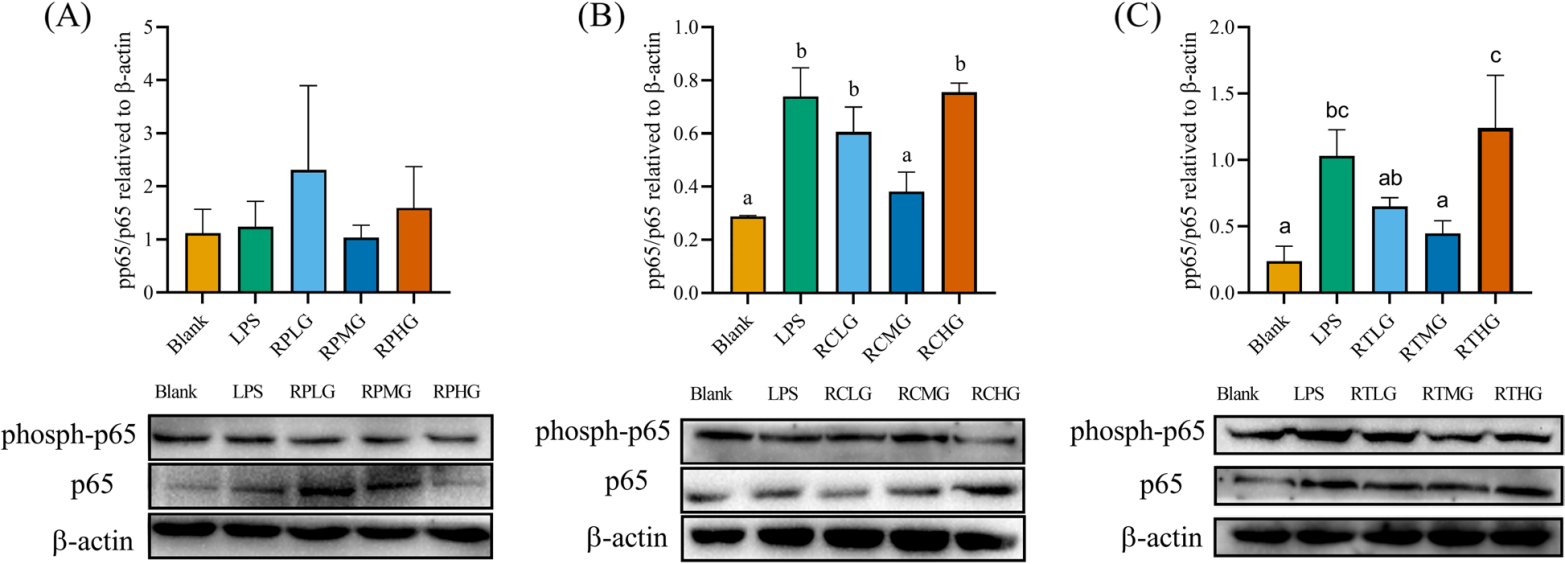
Effects of *L. plantarum* G83 on NF-κB/p65 protein phosphorylation level in LPS-induced RAW264.7 cells. The phosphorylation level of P65 protein was determined by western blot after RAW264.7 pre-treated (**A**), co-cultured (**B**) and post-treated with different viable count of *L. plantarum* G83. The same batch of different treatment samples ran on the same gel and blots closest to the mean abundance cropped from different gels were presented in the figure. All data are shown as mean ± SD. Data are analyzed with one-way ANOVA (Tukey's test). Different letters mean *p* < 0.05, same letter means *p* > 0.05

We further investigated inflammation-associated genes on the transcriptional level. Results showed that IL-6 gene was significantly decreased in all prevented treatment groups, while IL-10 gene was significantly promoted. The expression level of TLR4, NF-κB, MyD88, and iKKβ gene was also down-regulated in all three *L. plantarum* G83 pre-treatment groups (Fig. 10). In co-culture groups (Fig. 11), the expression of TNF-α was significantly decreased in the RCMG and RCHG groups. Meanwhile, IL-1β gene was promoted by the RCHG treatment group, and IL-10 gene showed a higher expression level in the RCLG treatment group. The TLR4 and NF-κB genes in all co-culture groups showed a lower expression level compared to that of the LPS treatment group. In addition, MyD88 and iKKβ genes also showed the same change trend. In *L. plantarum* G83 post-treatment groups (Fig. 12), the *L. plantarum* G83 showed a capability to inhibit the IL-1β, TNF-α, and IL-6 gene expression. However, the IL-10 gene was promoted in all *L. plantarum* G83 treatment groups. Consistent with the observation above, the TLR4, NF-κB, MyD88, and iKKβ genes were down-regulated by *L. plantarum* G83 in the treatment groups. Western blot results indicated that the phosphorylation of NF-κB/p65 was significantly decreased in the medium dose of the co-culture and treatment group (Fig. 13).

## Discussion

*Lactobacillus* spp. is an important indigenous bacterium in intestinal microbiota as well as one of the most widely used probiotics supplementary [24, 25]. It can initial non-immune and immune response to pathogen invasion and inflammation response in gastrointestinal tract [26, 27]. *Lactobacillus* spp. has been shown to decrease epithelial permeability through interaction with epithelial barrier. *Lactobacillus* spp. has been previously shown to influence the expression and localization of tight junction proteins [15, 28, 29]. Moreover, changes in the permeability of epithelial cells are also accompanied by an inflammatory response. Gratifyingly, there are various components on the surface of *Lactobacillus* spp., such as lipoteichoic acid (TLA), peptidoglycan, and cell surface protein (S-layer protein) [30, 31]. These components act as ligands to interact with PRRs on macrophages, dendritic cells [32], and epithelial cells [15] by triggering immune signal transduction. After being recognized by the receptors, ligands can activate immune signal pathways by producing cytokines and chemokines to interact with the immune system. In the present study, we demonstrated that *L. plantarum* G83 is not toxic to Caco-2 and RAW264.7 cells, and it showed an ability to increase the integrity of epithelial barrier in LPS-impaired Caco-2 and reduce pro-inflammatory response in RAW264.7 induced by LPS.

The physical barrier function of intestine is the first barrier for host against foreign materials invasion. Maintaining intercellular junction integrity is the prerequisite for exerting its barrier function [33]. Tight junction proteins are serviced as a gatekeeper for the paracellular pathway to keep intercellular junction integrity. As a gatekeeper between cells, TJs are threatened by pathogenic bacteria and proinflammatory mediators resulting in the development of inflammation and disease [34]. Thus, macromolecular substances from intestinal lumina can easily enter the host through the paracellular pathway when epithelial cell permeability is challenged by inflammation. In our study, the expression of Claudin-1, Occludin, and ZO-1 protein was decreased in Caco-2 monolayer cells after the LPS challenge. While the western blot and qRT-PCR results showed that ZO-1 and Occludin-1 were enhanced in medium dose of *L. plantarum* G83 co-culture and post-treatment group. Resta-Lenert reported that *L. acidophilus* ATCC4356 enhanced expression of ZO-1 and Occludin in HT-29 and Caco-2 monolayer cells models to reduce epithelial dysfunction caused by enteroinvasive *Escherichia coli* 029: NM [35, 36]. *L. rhamnosus* MTCC-5897 was demonstrated to compete with ETEC for binding sites on the surface of Caco-2, and restore ZO-1, Claudin-1, Occludin and Cingulin damage destroyed by ETEC [37]. Lower FITC-D4 fluorescence intensity in the lower compartment of the transwell plate in our result also concluded that the paracellular permeability was improved by *L. plantarum* G83. Many studies have shown that *Lactobacillus* spp. could enhance the paracellular permeability in LPS- or pathogen-impaired Caco-2 monolayer determined by FITC-D4 [14, 38].

Clayburgh concluded that TNF-α could increase the permeability of intestinal epithelial cells by enhancing the activity of myosin light chain kinase (MLCK) which will destroy intestinal tight connector protein. And the use of TNF-α antagonists can significantly restore intestinal barrier function [39]. Therefore, regulation of TNF-α can not only reduce intestinal inflammation but also restore intestinal barrier function. In this study, the expression of TNF-α decreased after *L. plantarum* G83 intervention, and the content of anti-inflammatory cytokine IL-10 also showed an increasing trend. It is continued to prove that *L. plantarum* G83 can strengthen the tight junctions of intestinal epithelial cells. Overall, these data demonstrate that *L. plantarum* G83 could be a good candidate to improve TJs in LPS-impaired Caco-2 cells.

Macrophages are effector cells of the innate immune system that phagocytose bacteria, foreign materials, and damaged cells. In addition, they can also present antigens to T cells and initiate inflammation by releasing cytokines that activate downstream immune responses [40]. Studies have shown that *Lactobacillus* spp. enhance the phagocytic function of macrophages and the presentation of antigenic substances in vivo and in vitro [41, 42]. Our neutral red phagocytosis testing result indicated that the phagocytic function of macrophages was enhanced after *L. plantarum* G83 treatment. Besides, TNF-α is the earliest synthesized cytokine in the inflammatory response, and IL-6 is the main response cytokine [43]. In this study, the inflammation was suppressed with a lower level of TNF-α and IL-6 in the cell culture supernatant of *L. plantarum* G83 co-culture and post-treatment groups. Meanwhile, only a mild anti-inflammatory effect of *L. plantarum* G83 on RAW264.7 cells was observed in the pre-treatment groups. It indicated that the sustained effect of a certain dose is an important condition for *L. plantarum* G83 as a biological agent to participate in the regulation of inflammation [44]. IL-10 is one of the main anti-inflammatory cytokines, which is crucial for maintaining the balance of inflammation and immunopathological response.

In the present study, the secretion of IL-10 was significantly decreased in the medium dose of *L. plantarum* G83 post-treatment group, and a similar result was observed in the low and high dose of co-culture group. However, the qRT-PCR result of IL-10 gene was opposite to this of the ELISA result, indicating that the IL-10 gene expression level increased after co-cultured or post-treated with *L. plantarum* G83. It is demonstrated that PBMC from patients with allergic inflammation co-cultured with exogenous IL-10 could significantly inhibit the production of cytokines such as IL-6, TNF-α, and IL-1β [45]. It is further proved that IL-10 plays an important role in the negative feedback regulation of inflammatory response by *L. plantarum* G83, and its mechanism of action needs to be further studied. In addition, the NO released in the cell supernatant had the same change trend as IL-10. As an important signal mediator, NO plays a dual role in inflammatory response. It can not only inhibit the activity of microbes inside and outside the cell but also mediate the cytotoxic response and activate T lymphocytes [46, 47].

There is a large variation in protein composition on the surface of different *Lactobacillus* strains. They can be recognized by different receptors on the host cell and activate the immune signal pathway. This may be one of the reasons why different strains have different probiotic profiles [48]. Many studies demonstrated that the *Lactobacillus* spp. regulates immune response by triggering off the TLR2 and TLR4 relative pathways to boost or inhibit pro-inflammatory cytokine release [18–20]. Direct exposure of inactivated *Lactobacillus* spp. to macrophages can activate the TLR2-NF-κB pathway and promote the expression of pro-inflammatory cytokines such as IL-8, TNF-α, IL-12p70, and IL-6 [49]. *L. plantarum* L15 showed to alleviate DSS-induced inflammation and down-regulate the expression of TLR4 and MyD88 genes as well as genes associated with NF-κB signaling pathway [50]. Results of our study showed that genes related to the TLR4/NF-κB signaling pathway were significantly down-regulated after *L. plantarum* G83 intervention. At the same time, the phosphorylation level of NF-κB/p65 was also significantly reduced in our study. However, we didn’t observe any change in TLR2 in any treatment groups. Therefore, we speculated *L. plantarum* G83 may not use TLR2 but TLR4 for signal transduction to interfere with the inflammatory response.

Because of the limitation of giant panda resources, there was little possibility to use panda-derived cells for experiments. Therefore, the widely used intestinal epithelial cell line (Caco-2) and immune cell line (RAW264.7) were used to reveal the mechanism of action of *L. plantarum* G83 in intestinal barrier and immune regulation in vitro [51, 52]. It is well known that probiotics show different properties in different hosts and even between different individuals, and different cell lines may have different testing results [53, 54]. Moreover, generally, the results of in vitro study may not be completely consistent with those of in vivo study. In other words, our findings may not all be validated in giant pandas. However, the results of current in vitro study could prove that *L. plantarum* G83 has no toxicity to cells and ability to alleviate inflammatory response. It also provides us with clues for further understanding on its mechanism of interaction with host to enhance tight junction proteins to strengthen the barrier function, and alleviate the inflammatory response induced by LPS in vivo. More in vitro and in vivo testing are needed to verify our current findings.

## Conclusion

In summary, the result from our study demonstrated that *L. plantarum* G83 possessed promotion of intercellular barrier function and improvement of inflammatory response in LPS induced inflammation model in vitro. Therefore, *L. plantarum* G83 is a promising candidate for anti-inflammation treatment in vitro.

## Materials and methods

### *L. plantarum* G83 strain and cell lines

The *L. plantarum* G83 (CCTCC M2016245) was isolated from giant panda feces, and it is available at China Center for Type Culture Collection (CCTCC, Wuhan, CHN). Murine derived macrophages cell line-RAW264.7 (GDC0143) and human colon adenocarcinoma cell line-Caco-2 cell line (GDC153) were purchased from CCTCC.

### Cell culture and cell viability assay

RAW264.7 cells were cultured in Dulbecco's Modified Eagle Medium (DMEM) (Hyclone, Logan, UT, USA) with 10% fetal bovine serum (FBS) (CellMax, Beijing, CHN), while the Caco-2 cells were cultured in RPMI 1640 instead. Cells were maintained at 37 °C, in a 5% CO_2_, 95% air atmosphere incubator. The cell counting kit-8 (CCK-8) (BOSTER, Wuhan, CHN) was used to measure the viability of cells. Briefly, 1.0 × 10^4^ cells were seeded in a 96-wells plate and after overnight incubation, cells were treated with 1.0 × 10^4^, 1.0 × 10^5^, 1.0 × 10^6^ and 1.0 × 10^7^ CFU of *L. plantarum* G83 for 8 h individually. After that, cells were washed with PBS and replaced medium with fresh DMEM, 10 µl of CCK-8 solution was added to each well and incubated for another 1 h. The optical density (OD) was measured using Varioskan™ LUX multimode microplate reader (ThermoFisher, Waltham, MA, USA) at 450 nm.

### Effects of *L. plantarum* G83 on LPS-impaired tight junctions in Caco-2 cells

#### Caco-2 cell monolayer

5.0 × 10^4^ of Caco-2 cells seeded on the top insert of the transwell plate (24-well plate, polystyrene membrane, 0.4 μm pore size). The transmembrane electrical resistance (TEER) between the upper and lower chambers of the transwell is measured using Millicell Res-2 cell resistance meter (MilliporeSigma, Burlington, MA, USA) until the TEER is stable. All Caco-2 cell monolayer cultures followed the time determined by Millicell Res-2 cell resistance meter mentioned above.

#### Paracellular permeability and tight junction proteins determination in Caco-2 cell monolayer

Caco-2 cultured on the top insert of the transwell plate and formed a monolayer as mentioned above. While another batch of Caco-2 cell monolayer was cultured on the 6-well plate. Treatment groups are set as follows:

A), Cells were pre-treated with different concentrations of *L. plantarum* G83 for 8 h, 0 CFU (LPS group), 1.0 × 10^6^ CFU (CPLG group), 1.0 × 10^7^ CFU (CPMG group), 1.0 × 10^8^ CFU (CPHG group), after washing away the *L. plantarum* G83 followed LPS (1 μg/ml) stimulation for another 8 h; B), cells were co-cultured with LPS (1 μg/ml) and different concentration of *L. plantarum* G83 at the same time for 8 h, 0 CFU + LPS (LPS group), 1.0 × 10^6^ CFU + LPS (CCLG group), 1.0 × 10^7^ CFU + LPS (CCMG group), 1.0 × 10^8^ CFU + LPS (CCHG group); C), after stimulation with LPS (1 μg/ml) for 8 h, cells were treated with different concentration of *L. plantarum* G83 for 8 h after washed with PBS for 5 times, 0 CFU (LPS group), 1.0 × 10^6^ CFU (CTLG group), 1.0 × 10^7^ CFU (CTMG group) and 1.0 × 10^8^ CFU (CTHG group). Untreated cells served as a blank (Blank group) added in each group.

For cells cultured on a transwell plate, the insert and bottom well were washed with PBS. 100 μl of 1 mg/mL FITC-D4 in Hank’s solution was added in the insert and incubated in the 5% CO_2_ incubator at 37 ℃ for 1 h. After incubation, 100 μl solution from the lower compartment of the plate was used to measure fluorescence intensity under 492 nm as excitation wavelength and 520 nm as emission wavelength. All trials were carried out in six repeats.

For cells cultured on 6-well plate, after completing the treatments above, the supernatant was collected for cytokines analysis, and cells were lysed for protein and gene extraction. All trials were carried out in triplicate.

### Effects of *L. plantarum* G83 on LPS-induced RAW264.7 cells

RAW264.7 cells (1.0 × 10^6^/well) were seeded on a 6-well plate, after overnight incubation, cells were washed 5 times with PBS. After that, treatments were performed as same as that on Caco-2 cells. Treatment groups are set as follows:

A), cells were pre-treated with different concentrations of *L. plantarum* G83 for 8 h, 0 CFU (LPS group), 1.0 × 10^6^ CFU (RPLG group), 1.0 × 10^7^ CFU (RPMG group) and 1.0 × 10^8^ CFU (RPHG group), after washing away the *L. plantarum* G83 followed another 8 h stimulation by LPS (1 μg/ml) was performed; B), Cells were co-cultured with LPS (1 μg/ml) and different abundance of *L. plantarum* G83 for 8 h, 0 CFU + LPS (LPS group), 1.0 × 10^6^ CFU + LPS (RCLG group), 1.0 × 10^7^ CFU + LPS (RCMG group), 1.0 × 10^8^ CFU + LPS (RCHG group); C), after stimulation with LPS (1 μg/ml) for 8 h, cells were treated with different concentrations of *L. plantarum* G83 for 8 h after washed with PBS for 5 times, 0 CFU (LPS group), 1.0 × 10^6^ CFU (RTLG group), 1.0 × 10^7^ CFU (RTMG group) and 1.0 × 10^8^ CFU (RTHG group). Untreated cells served as a blank (Blank group) added in each group. After completing the treatment above, the supernatant was collected for cytokines analysis, and cells were lysed for protein and gene extraction. All trials were carried out in triplicate.

### Effects of *L. plantarum* G83 on macrophage phagocytosis

1.0 × 10^4^ cells were seeded to the 96-well plate, and the grouping and processing methods were as same as described above. Especially, different bacteria viable counts were used to maintain a consistent infection ratio, 1.0 × 10^4^ CFU (low-dose group), 1.0 × 10^5^ CFU (medium-dose group), and 1.0 × 10^6^ CFU (high-dose group) respectively. After the treatment, aspirate the supernatant and wash the cells 5 times with PBS, then 200 μl of 0.05% neutral red staining solution was added to each well. After incubation for 2 h, wash the cell 5 times in PBS and add 200 μl of cold cell lysate (glacial acetic acid: absolute ethanol = 1v:1v) to each well keeping at 4 °C overnight. Thoroughly mixed lysate and measure OD value at 540 nm.

### Enzyme-linked immunosorbent assay (ELISA)

The concentration of TNF-α, IL-6, IL-10, and NO in the supernatant of RAW264.7 culture supernatant was determined by ELISA kit (MLbio, Shanghai, CHN). While only that of TNF-α and IL-10 in the supernatant of Caco-2 culture was determined. The OD was detected by Varioskan™ LUX multimode microplate reader (Thermosphere, Waltham, MA, USA) at 450 nm.

### Western blot

The protein lysate was obtained from culture cells by culture cell total protein extraction reagent (BOSTER, Wuhan, CHN) with protease inhibitor and phosphatase inhibitor cocktail. Protein concentration was determined by a BCA protein analysis kit (BIOMED, Beijing, CHN). Primary antibodies included rabbit anti-β-actin (#20536-1-AP; Proteintech, Wuhan, CHN), rabbit anti-ZO-1 (#21773-1-AP; Proteintech, Wuhan, CHN), rabbit anti-Occludin (#27260–1-AP; Proteintech, Wuhan, CHN), rabbit anti-Claudin 1 (#bs-1428R; Bioss, Beijing, CHN), rabbit anti-NF-κB P65 (#bs-0465R; Bioss, Beijing, CHN) and rabbit anti-phospho-NF-κB p65 (#3033 T; CST, Danvers, MA USA). Goat anti-rabbit (#bs-0296G; Bioss, Beijing, CHN) was used as secondary antibody.

### RNA extraction and Real-Time quantitative PCR (RT-qPCR)

Total RNA was isolated from culture cells using TRIzol Reagent (Invitrogen, Carlsbad, CA, USA). The first-strand cDNA was synthesized from total RNA using RevertAid First Strand cDNA Synthesis Kit (ThermoFisher, Waltham, MA, USA). The RT-qPCR was performed using iTaq™ Universal SYBR Green (Bio-Rad, Hercules, CA, USA) on the CFX Connect™ Real-Time PCR Detection System (Bio-Rad, Hercules, CA, USA). The result of relative gene expression was normalized to that of β-actin and evaluated through the 2^−△△Ct^ method.

### Data analysis

All data were expressed as means with standard error. Statistical analysis was performed by one-way ANOVA in SPSS 19.0. Statistical significance was set at *p<*0.05.

### Supplementary Information

**Additional file 1.**

## Data Availability

All data generated or analyzed during this study are included in this published article and its Supplementary information files.
