# Supplementary material for: The panda-derived Lactiplantibacillus plantarum BSG201683 improves LPS-induced intestinal inflammation and epithelial barrier disruption in vitro
Source: BMC Microbiol. 2023 Sep 6;23:249. doi: 10.1186/s12866-023-02928-4 (PMC10481503; doi:10.1186/s12866-023-02928-4)

Content

Page2-20: Orginal Figure13A

2-7: β-actin
8-13: NF-kB
14-20: p-NF-kB

Page21-42: Original Figure13B

21-30: β-actin
31-36: NF-kB
37-42: p-NF-kB

Page43-62: Original Figure13C
43-50: β-actin
51-56: NF-kB
57-62: p-NF-kB

Original Figure13A-1(β-actin)

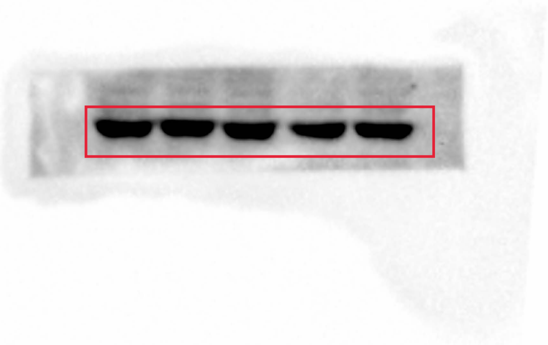

Original Figure13A-1 merged with white-light-field-of-view (WL-FOV) (β-actin)

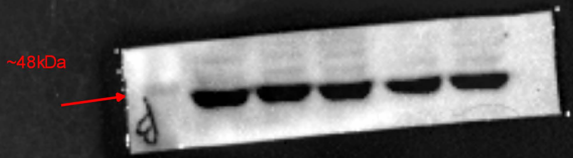

Original Figure13A-2 (β-actin)

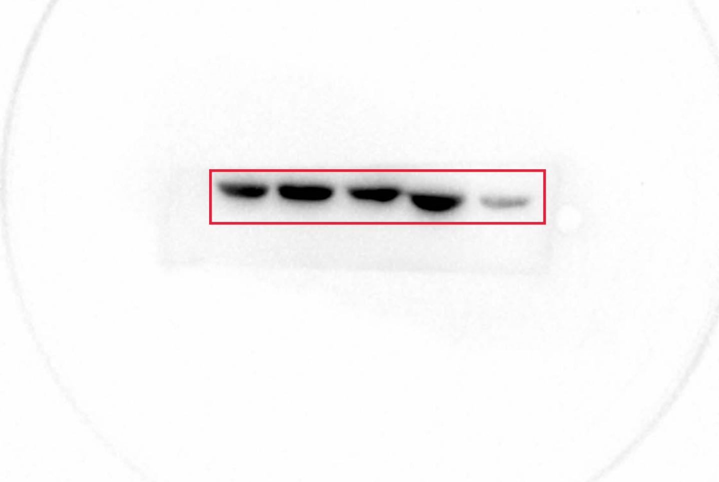

Original Figure13A-2 merged with WL-FOV (β-actin)

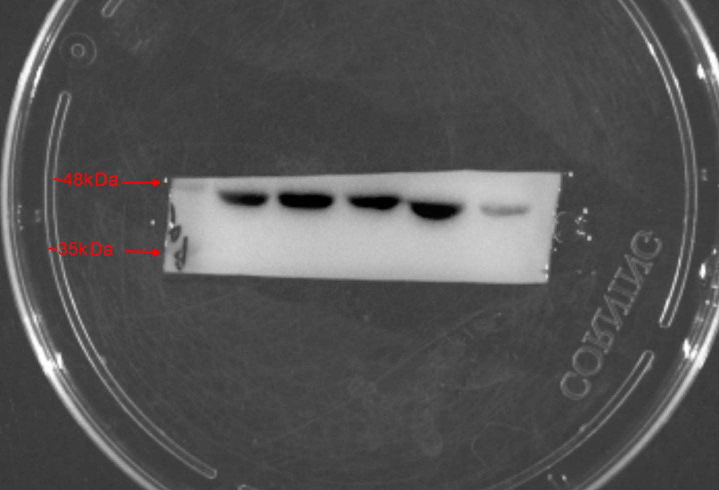

Original Figure13A-3 (β-actin)

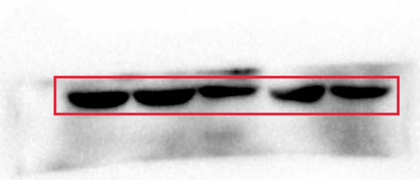

Original Figure13A-3 WL-FOV (β-actin)

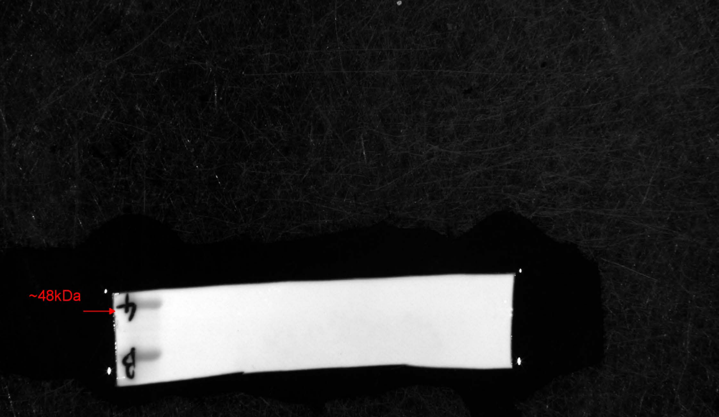

The two images cannot be merged in situ, so the original WL-FOV image is provided.

Original Figure13A-4 (NF-kB)

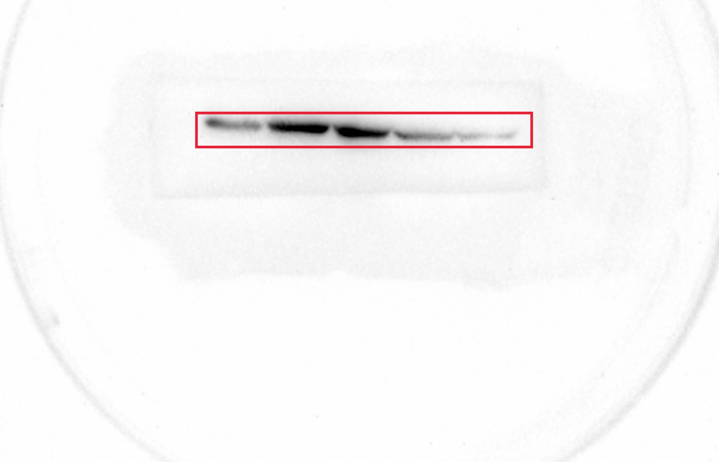

Original Figure13A-4 merged with WL-FOV (NF-kB)

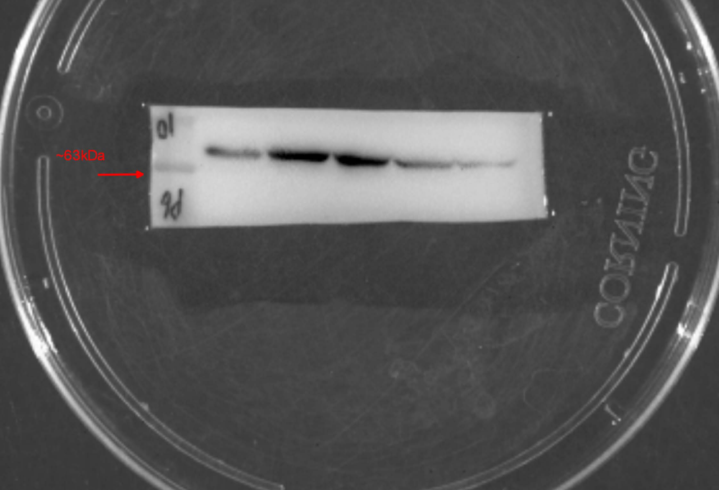

Original Figure13A-5 (NF-kB)

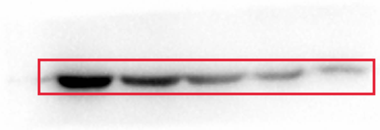

Original Figure13A-5 merged with WL-FOV (NF-kB)

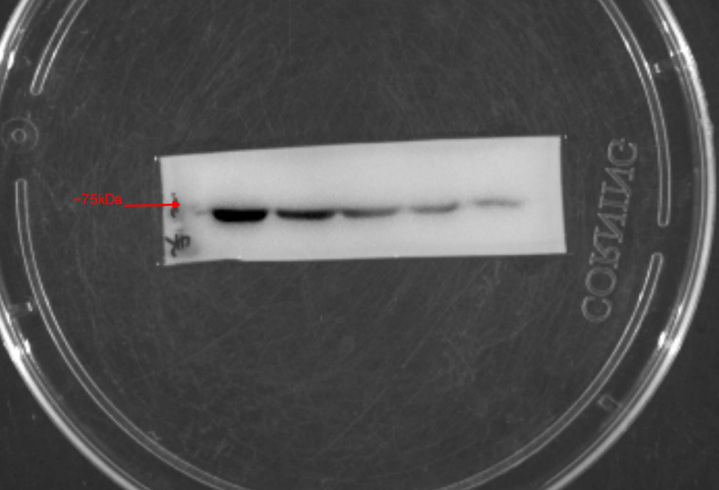

Original Figure13A-6 (NF-kB)

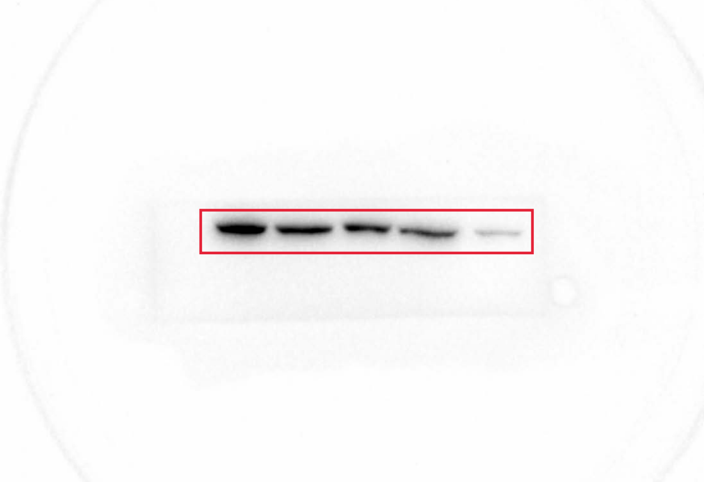

Original Figure13A-6 merged with WL-FOV (NF-kB)

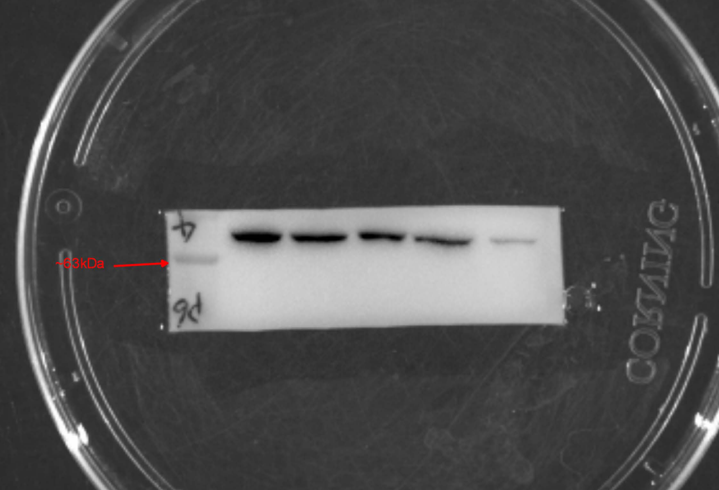

Original Figure13A-7 (p-NF-kB)

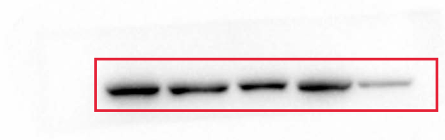

Original Figure13A-7 merged with WL-FOV (p-NF-kB)

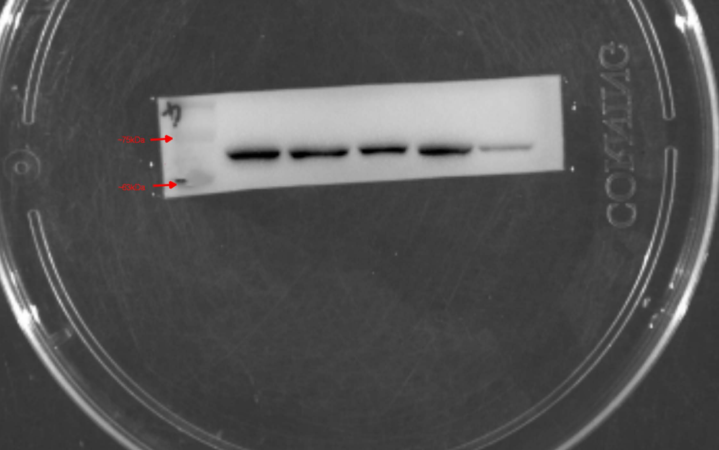

Original Figure13A-8-exposure1 (p-NF-kB)

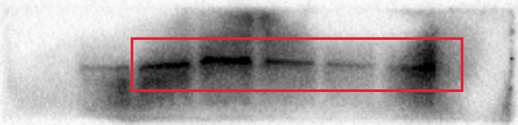

Since the WL-FOV image of this film is missing, three images with different exposure times are provided

Original Figure13A-8-exposure2 (p-NF-kB)

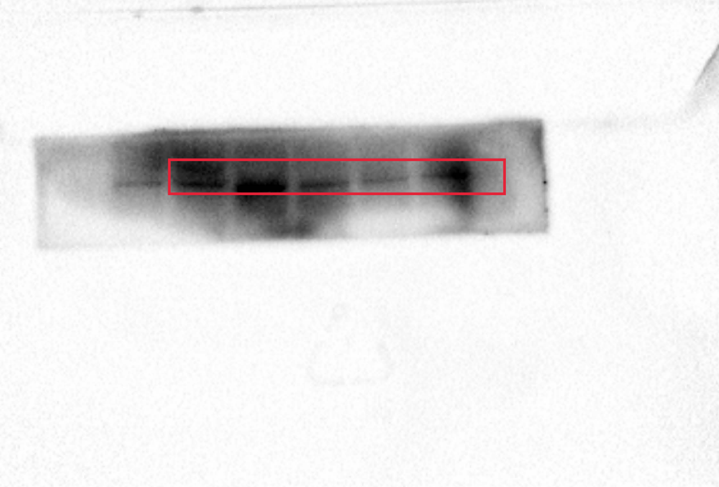

Original Figure13A-8-exposure3 (p-NF-kB)

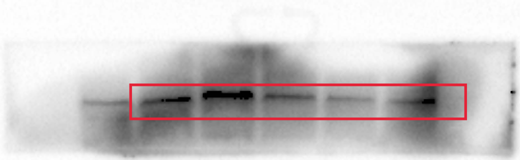

Original Figure13A-9 (p-NF-kB)

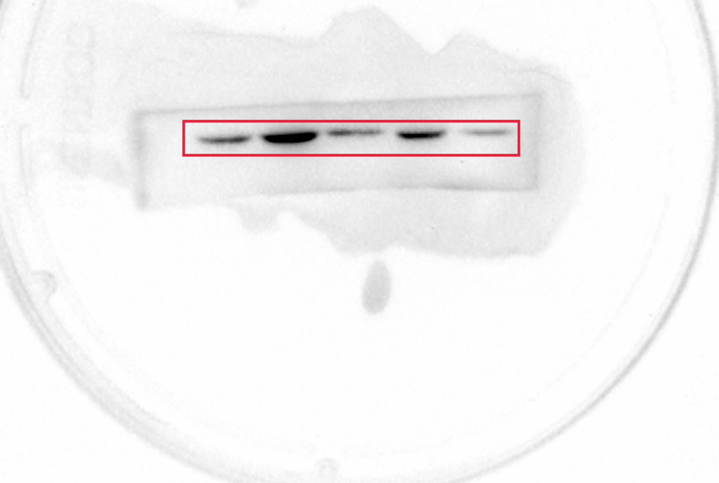

Original Figure13A-9 merged with WL-FOV (p-NF-kB)

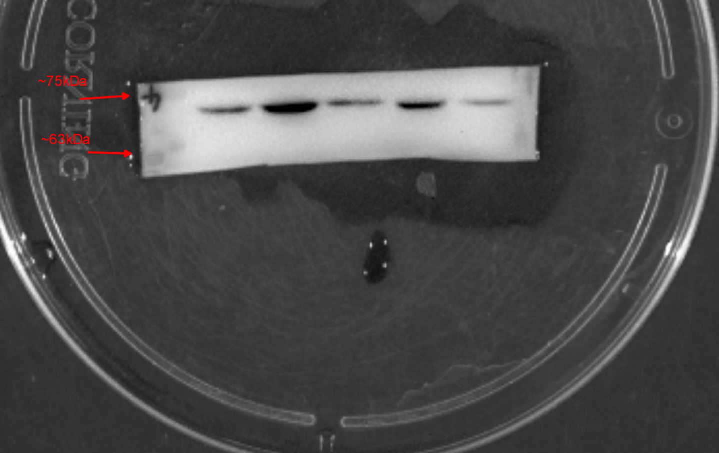

Original Figure13B-1 (β-actin)

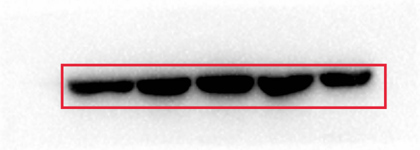

Original Figure13B-1 merged with WL-FOV (β-actin)

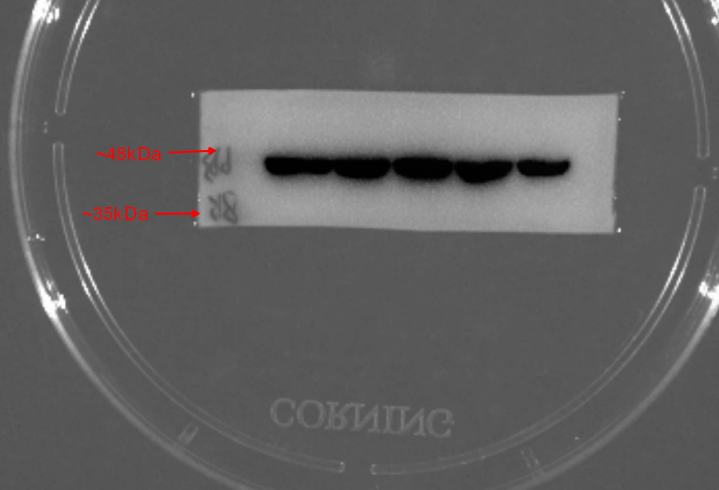

Original Figure13B-2 (β-actin)

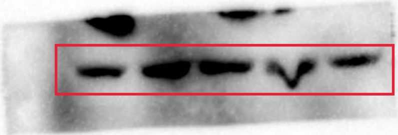

Original Figure13B-2 merged with WL-FOV (β-actin)

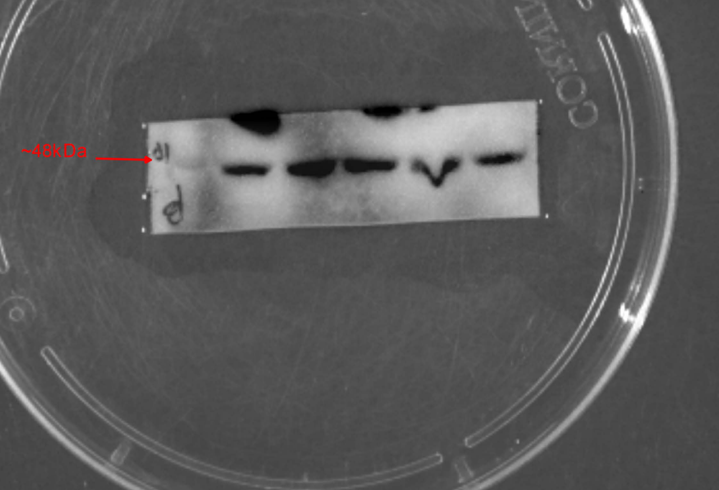

Original Figure13B-3 (β-actin)

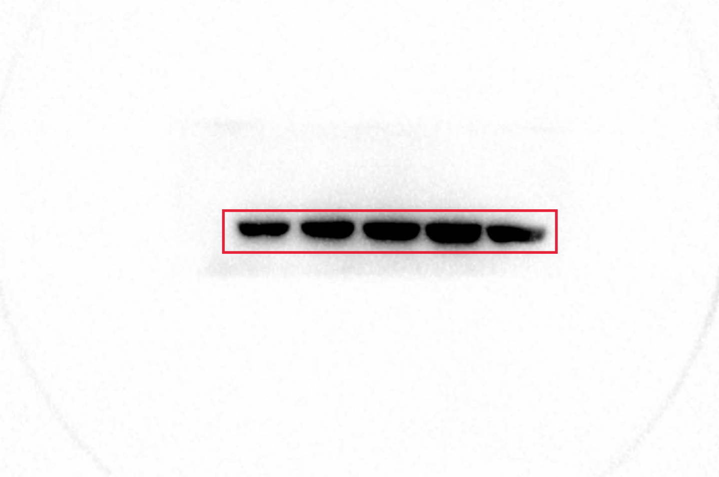

Original Figure13B-3 merged with WL-FOV (β-actin)

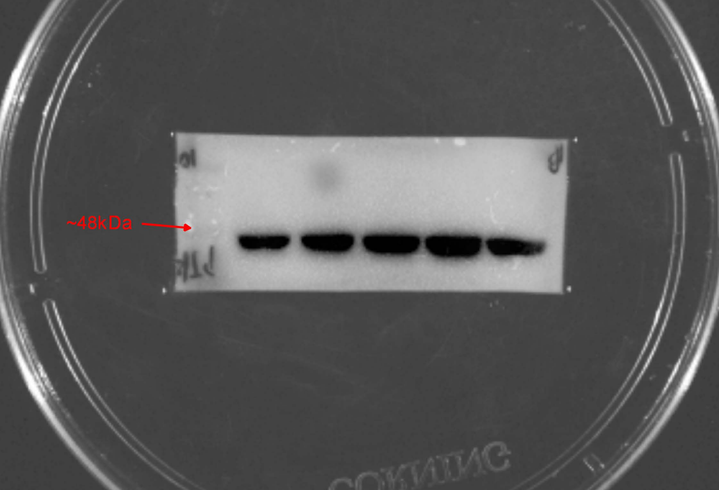

Original Figure13B-4 (β -actin)

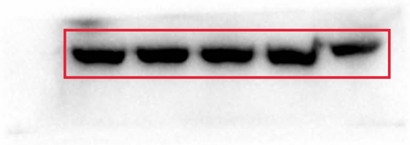

Original Figure13B-4 merged with WL-FOV (β-actin)

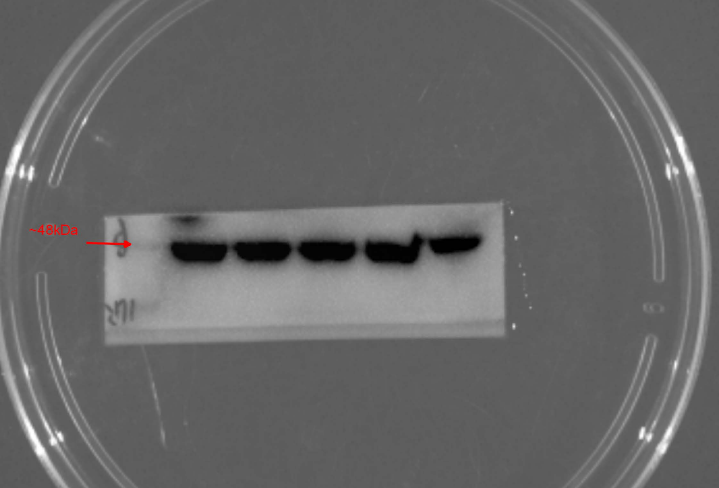

Original Figure13B-4 (β-actin)

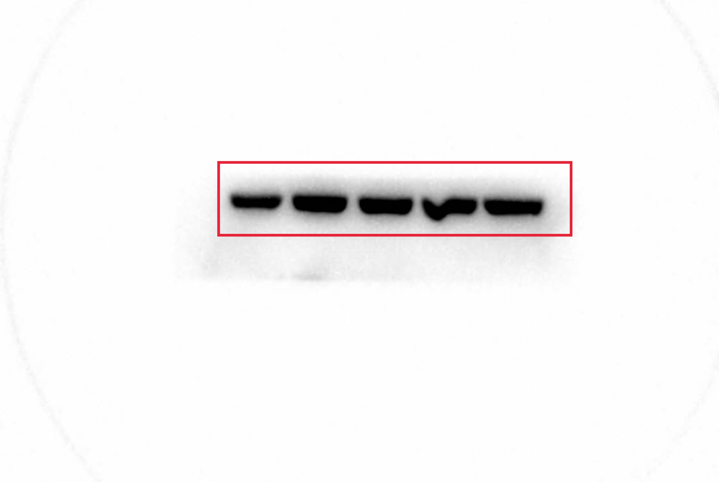

Original Figure13B-4 merged with WL-FOV (β-actin)

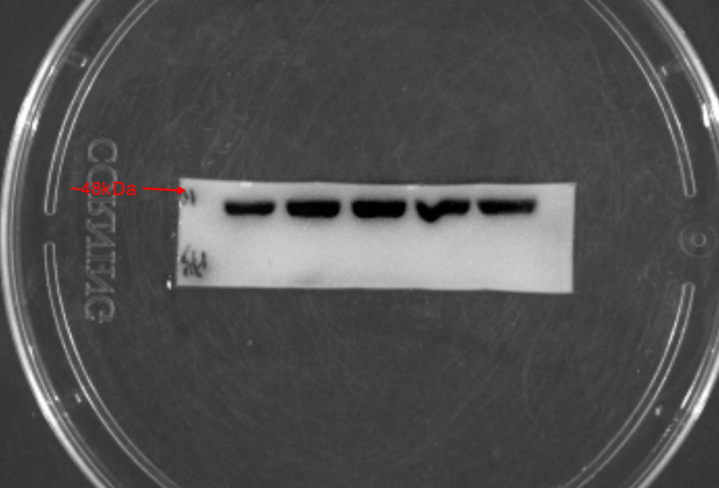

Original Figure13B-5-exposure1 (NF-kB)

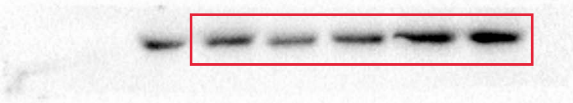

Since the WL-FOV image of this film is missing, two images with different exposure times are provided

Original Figure13B-5-exposure2 (NF-kB)

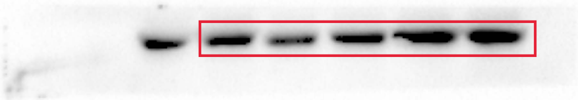

Original Figure13B-6 (NF-kB)

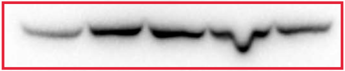

Original Figure13B-6 merged with WL-FOV (NF-kB)

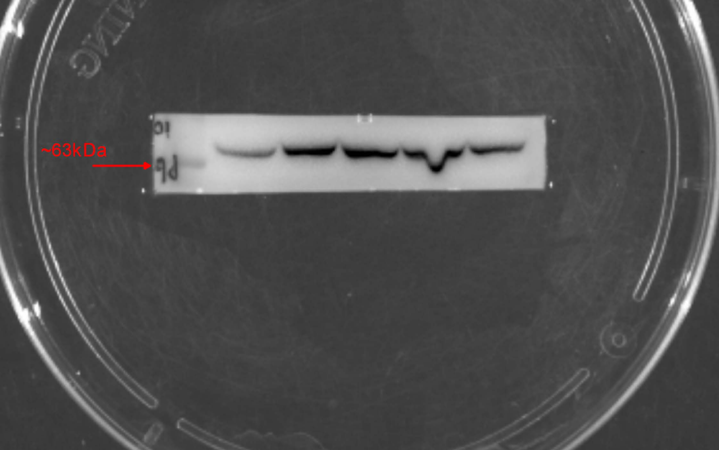

Original Figure13B-7 (NF-kB)

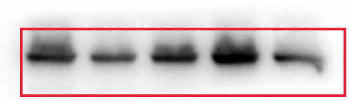

Original Figure13B-7 merged with WL-FOV (NF-kB)

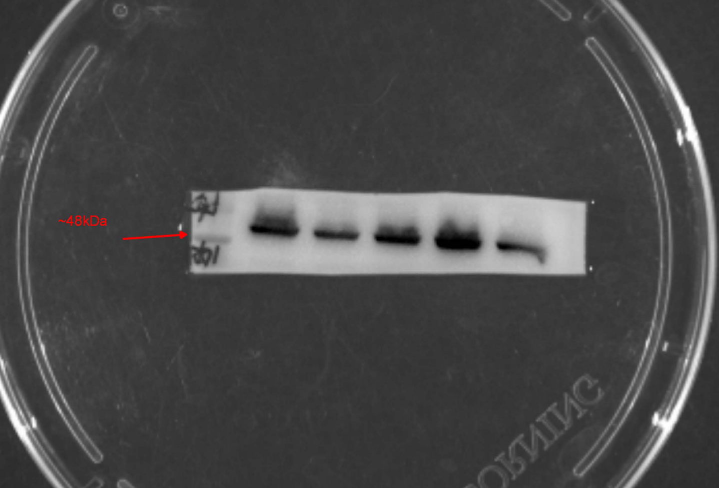

Original Figure13B-8 (p-NF-kB)

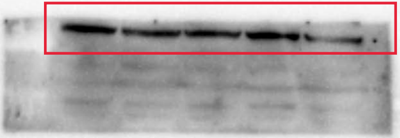

Original Figure13B-8 merged with WL-FOV (p-NF-kB)

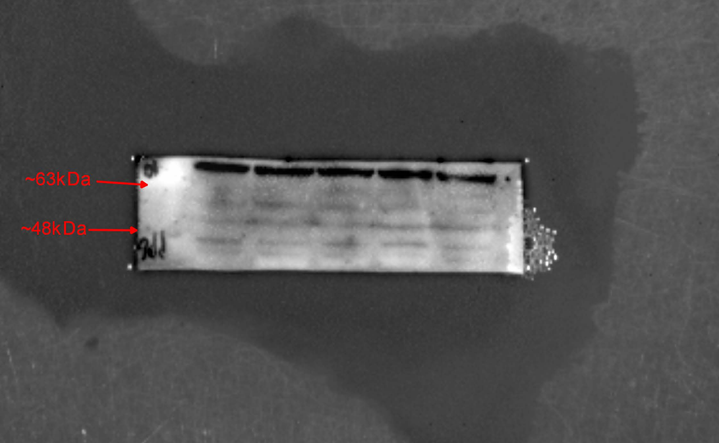

Original Figure13B-9 (p-NF-kB)

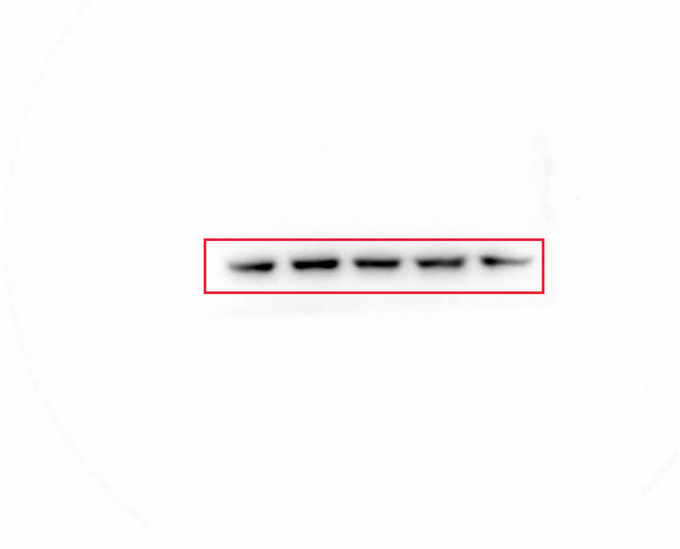

Original Figure13B-9 merged with WL-FOV (p-NF-kB)

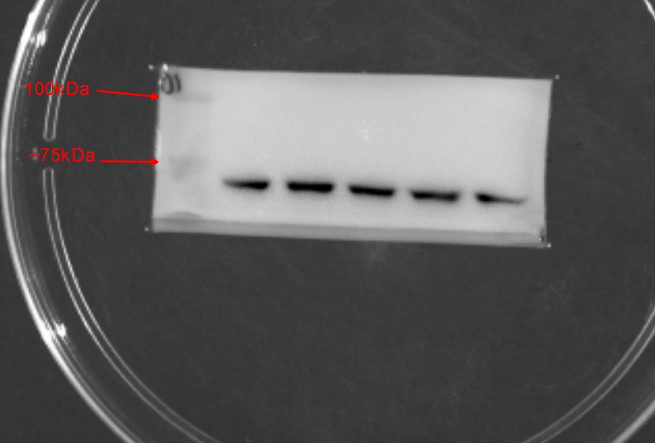

Original Figure13B-10 (p-NF-kB)

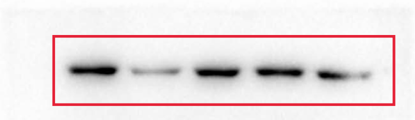

Original Figure13B-10 merged with WL-FOV (p-NF-kB)

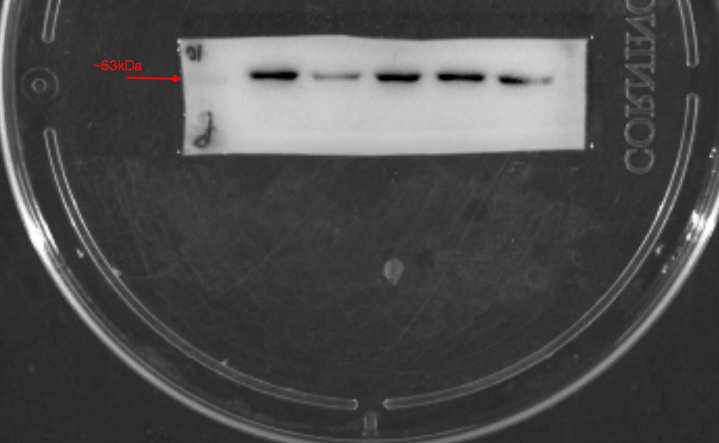

Original Figure13C-1 (β-actin)

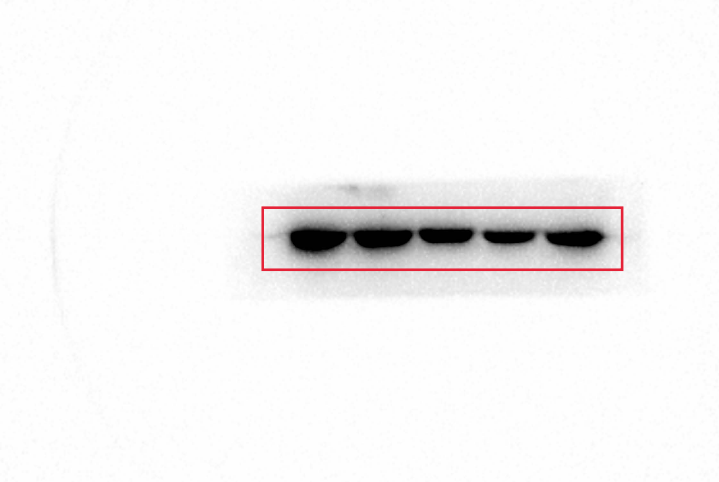

Original Figure13C-1 merged with WL-FOV (β-actin)

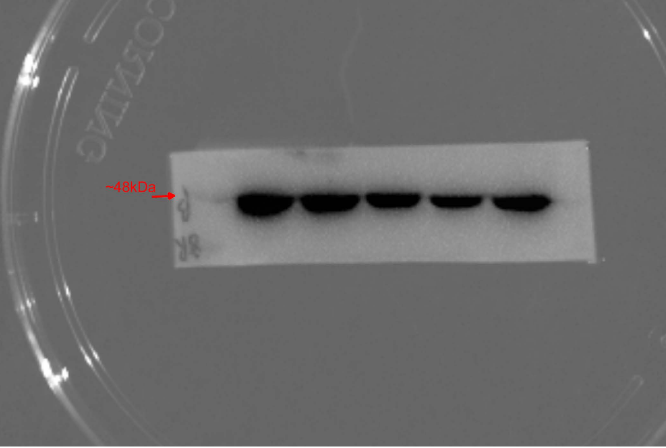

Original Figure13C-2 (β-actin)

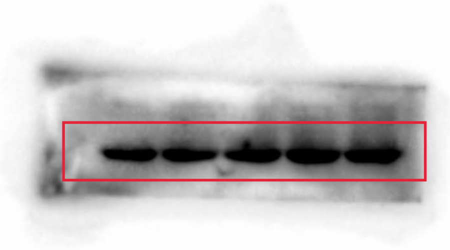

Original Figure13C-2 merged with WL-FOV (β-actin)

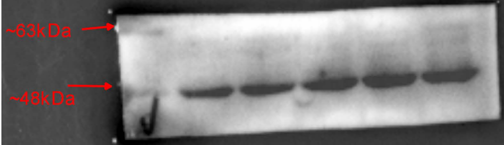

Original Figure13C-3 (β-actin)

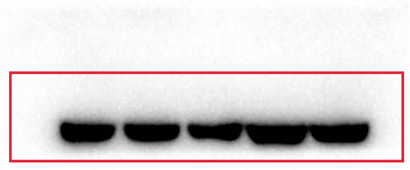

Original Figure13C-3 merged with WL-FOV (β-actin)

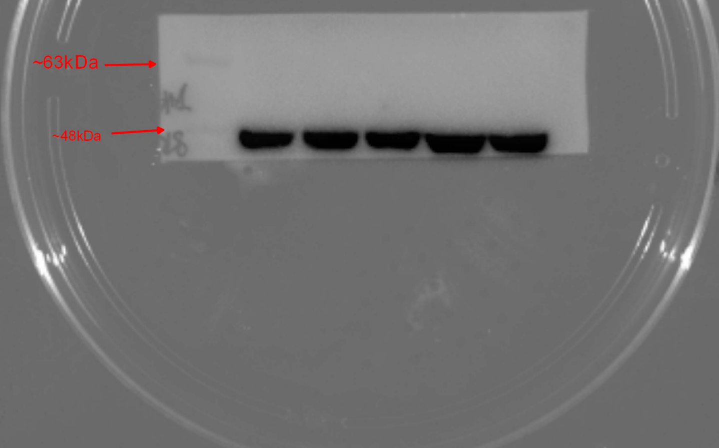

Original Figure13C-4 (β-actin)

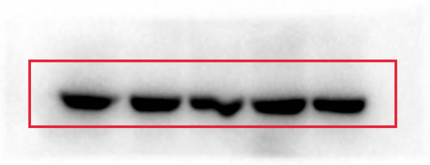

Original Figure13C-4 merged with WL-FOV (β-actin)

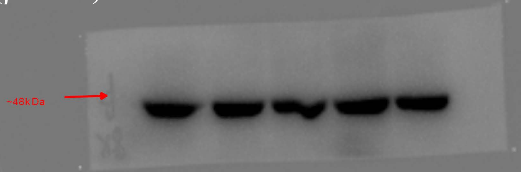

Original Figure13C-5 (NF-kB)

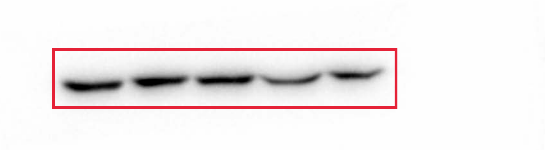

Original Figure13C-5 merged with WL-FOV (NF-kB)

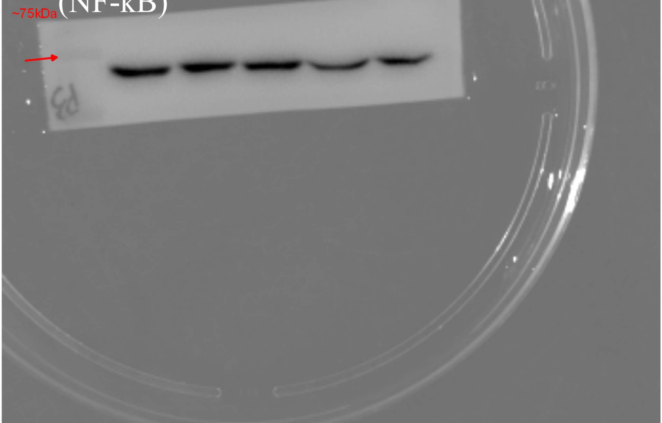

Original Figure13C-6 (NF-kB)

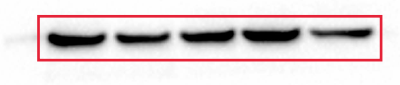

Original Figure13C-6 merged with WL-FOV (NF-kB)

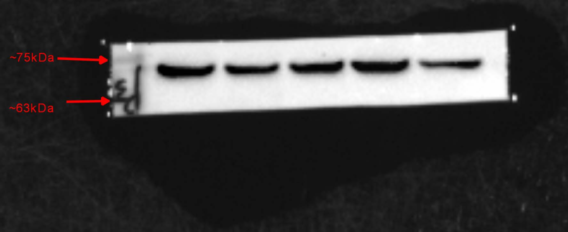

Original Figure13C-7 (NF-kB)

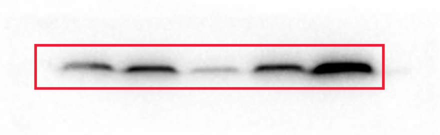

Original Figure13C-7 merged with WL-FOV (NF-kB)

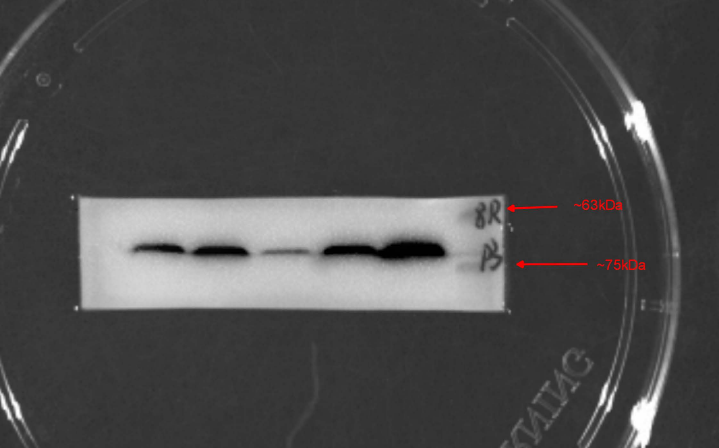

Original Figure13C-8 (p-NF-kB)

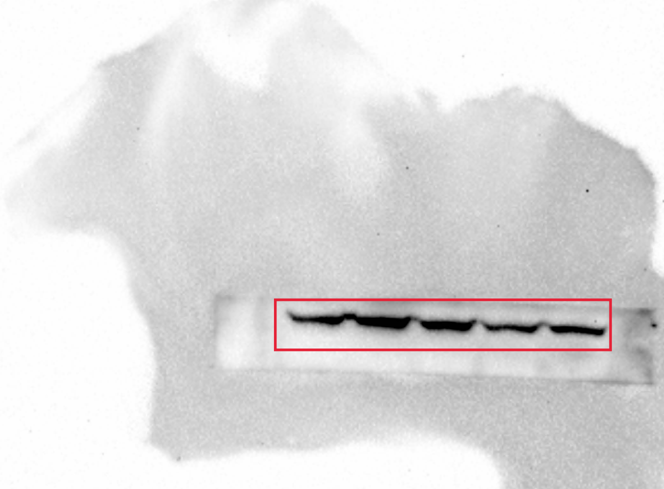

Original Figure13C-8 merged with WL-FOV (p-NF-kB)

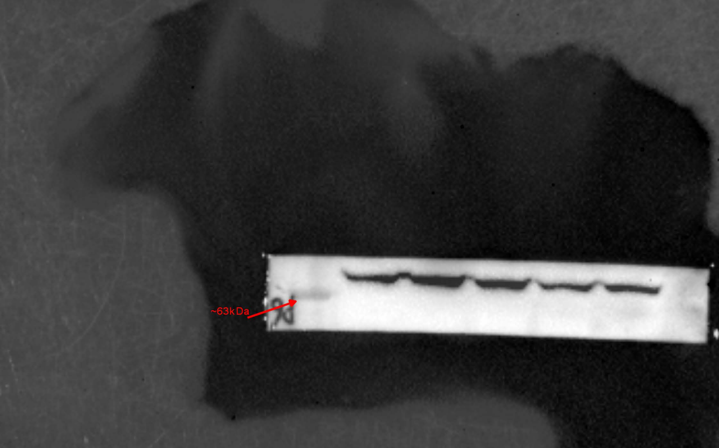

Original Figure13C-9 (p-NF-kB)

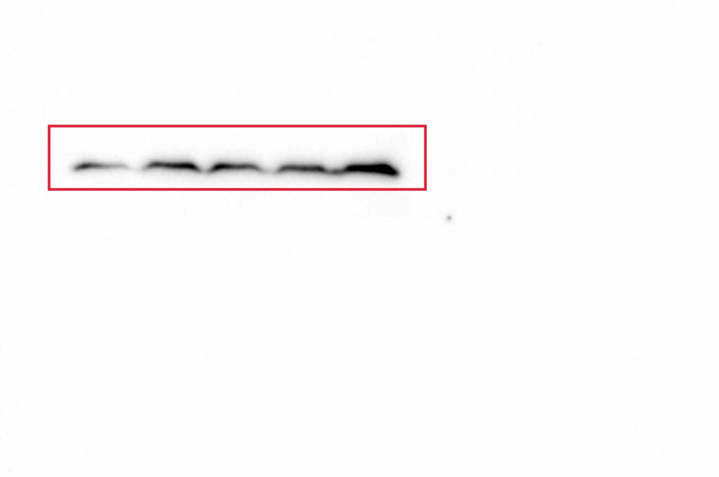

Original Figure13C-9 merged with WL-FOV (p-NF-kB)

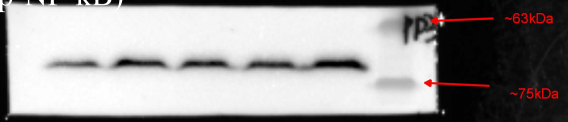

Original Figure13C-10 (p-NF-kB)

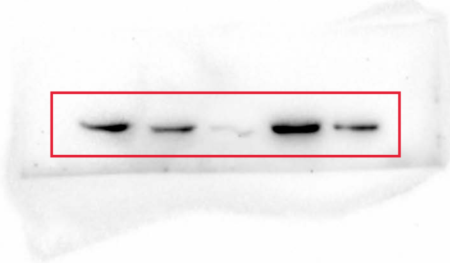

Original Figure13C-10 merged with WL-FOV (p-NF-kB)

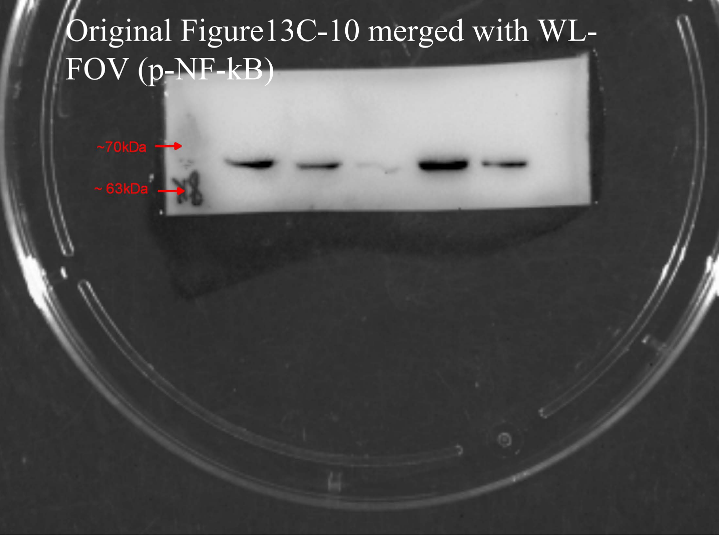

Supplement: Supplementary file 1 — Additional file 1. [file 12866_2023_2928_MOESM1_ESM.zip › original-images-figure13.pdf]
