# Supplementary material for: The panda-derived Lactiplantibacillus plantarum BSG201683 improves LPS-induced intestinal inflammation and epithelial barrier disruption in vitro
Source: BMC Microbiol. 2023 Sep 6;23:249. doi: 10.1186/s12866-023-02928-4 (PMC10481503; doi:10.1186/s12866-023-02928-4)

Content

pre-treatment group

Page2-25: Original Figure5A, 5D, 5G
2-7: Original Figure5A, 5D, 5G (β-actin)
8-13: Original Figure5A (Claudin-1)
14-19: Original Figure5D (Occludin-1)
20-25: Original Figure5G (ZO-1)

co-treatment group

Page26-49: Original Figure5B, 5E, 5H
26-31: Original Figure5B, 5E, 5H(β-actin)
32-37: Original Figure5B (Claudin-1)
38-43: Original Figure5E (Occludin-1)
44-49: Original Figure5H (ZO-1)

post-treatment group

Page50-73: Original Figure5C, 5F, 5I
50-55: Original Figure5C, 5F, 5H(β-actin)
56-61: Original Figure5C (Claudin-1)
62-67: Original Figure5F (Occludin-1)
68-73: Original Figure5I (ZO-1)

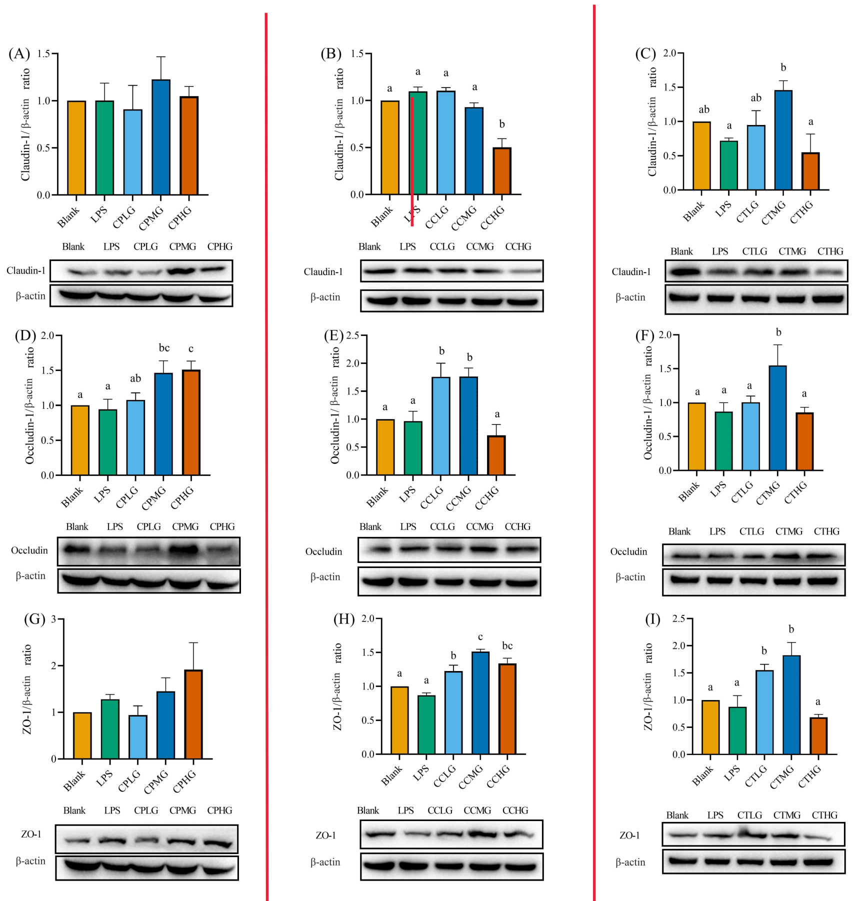

pre-treatment group co-treatment group post-treatment group

Original Figure5A-1, 5D-1, 5G-1 (β-actin)

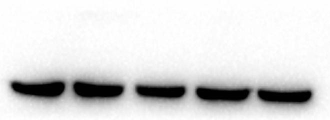

Original Figure5A-1, 5D-1, 5G-1 merged with white-light-fieled-of-view (WL-FOV) (β-actin)

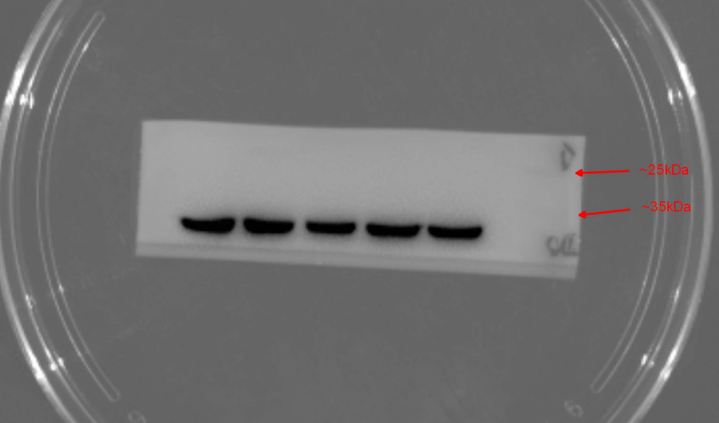

Original Figure5A-2, 5D-2, 5G-2 (β-actin)

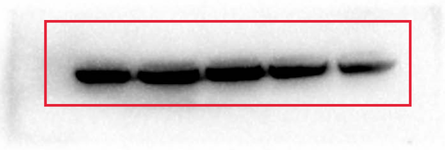

Original Figure5A-2, 5D-2, 5G-2 merged with WL-FOV (β-actin)

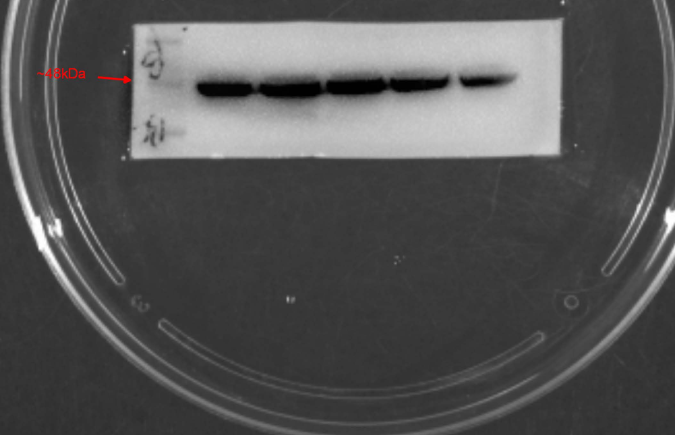

Original Figure5A-3, 5D-3, 5G-3 (β-actin)

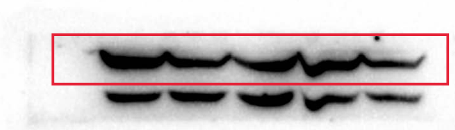

Original Figure5A-3, 5D-3, 5G-3 merged with WL-FOV (β-actin)

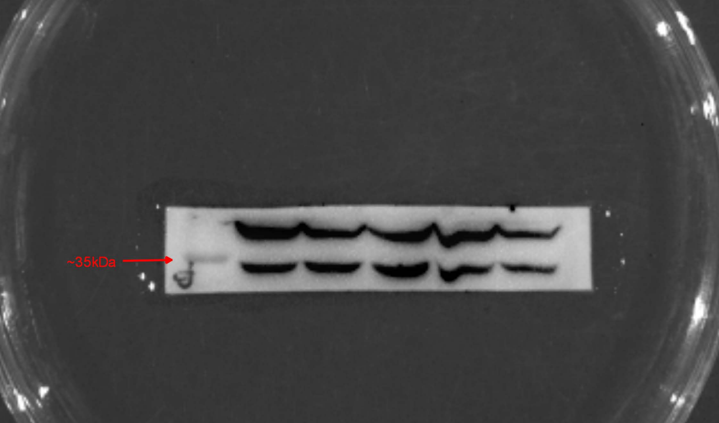

Original Figure5A-4 (Claudin-1)

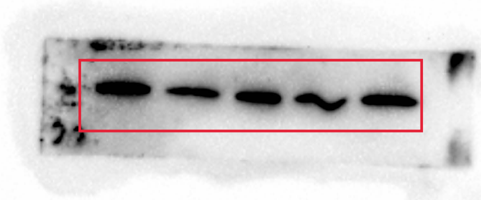

Original Figure5A-4 merged with WL-FOV (Claudin-1)

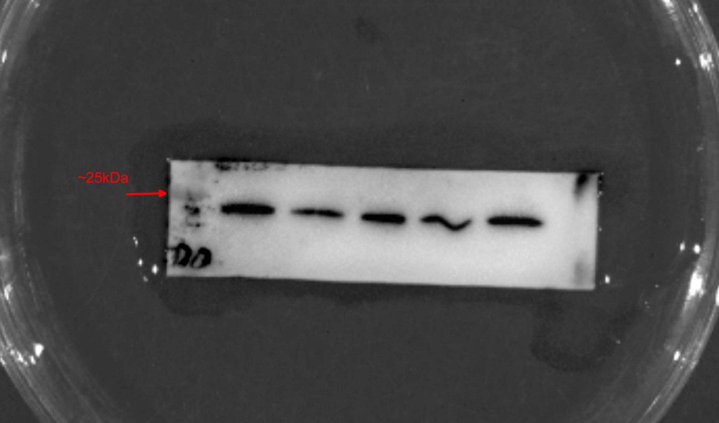

Original Figure5A-5 (Claudin-1)

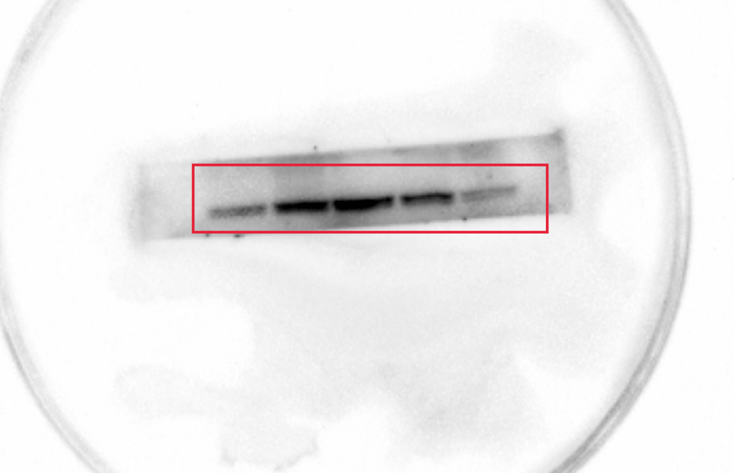

Original Figure5A-5 merged with WL-FOV (Claudin-1)

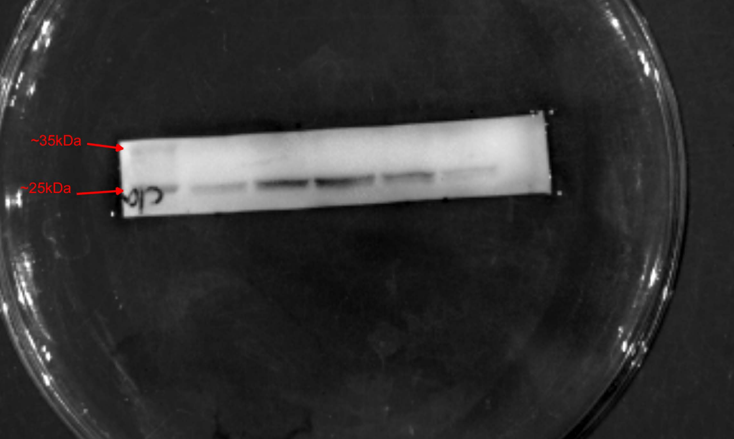

Original Figure5A-6 (Claudin-1)

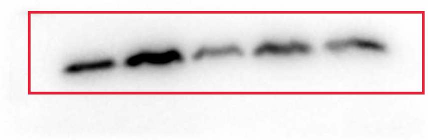

Original Figure5A-6 merged with WL-FOV (Claudin-1)

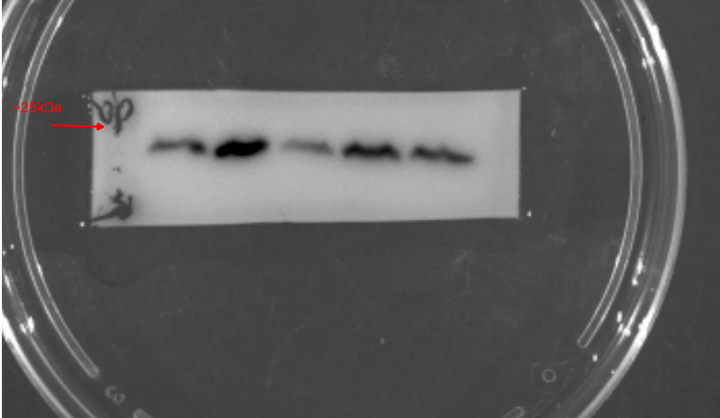

Original Figure5D-4 (Occludin)

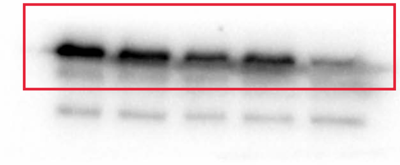

Original Figure5D-4 merged with WL-FOV (Occludin)

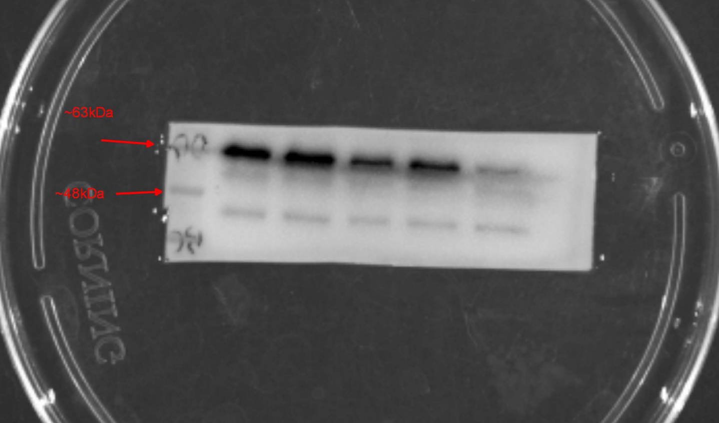

Original Figure5D-5 (Occludin)

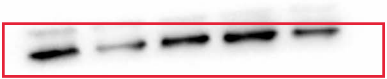

Original Figure5D-5 merged with WL-FOV (Occludin)

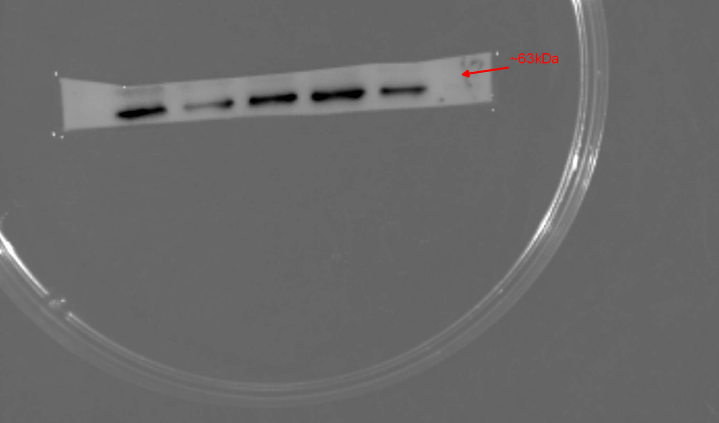

Original Figure5D-6 (Occludin)

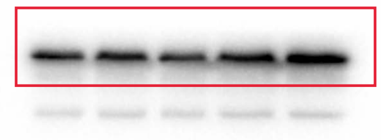

Original Figure5D-6 merged with WL-FOV (Occludin)

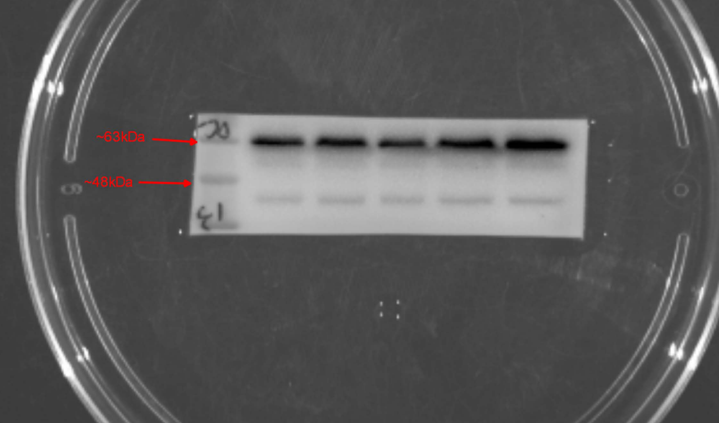

Original Figure5G-4 (ZO-1)

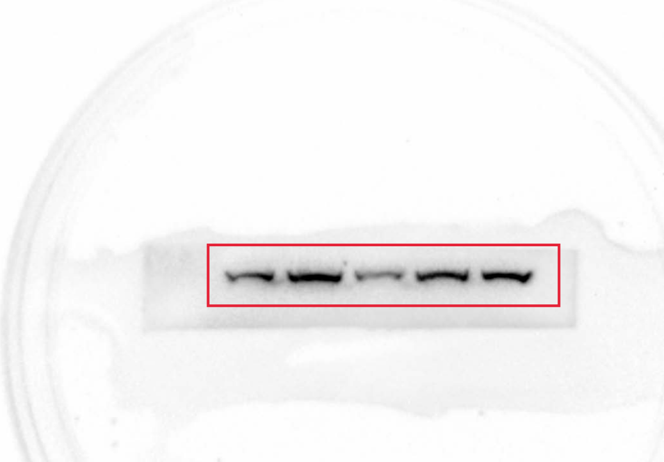

Original Figure5G-4 merged with WL-FOV (ZO-1)

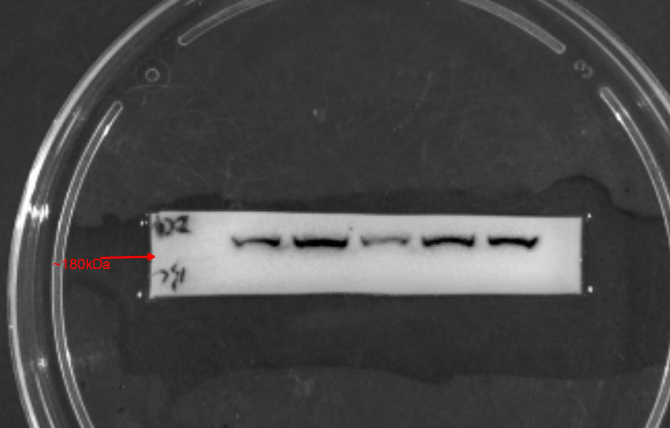

Original Figure5G-5 (ZO-1)

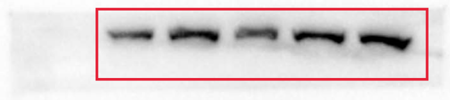

Original Figure5D-5 merged with WL-FOV (ZO-1)

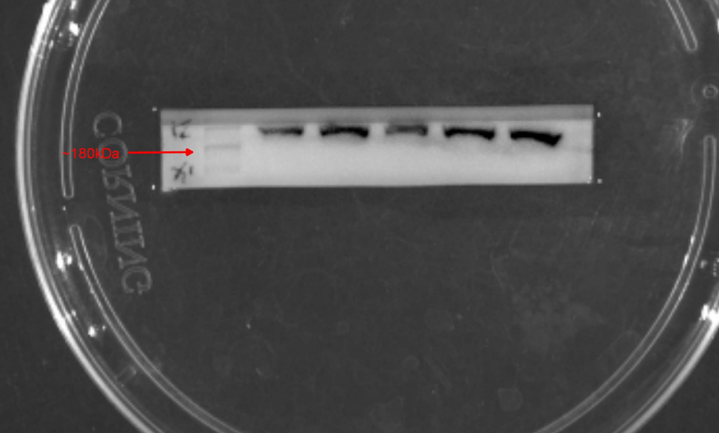

Original Figure5G-6 (ZO-1)

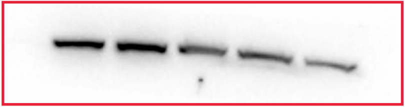

Original Figure5D-6 merged with WL-FOV (ZO-1)

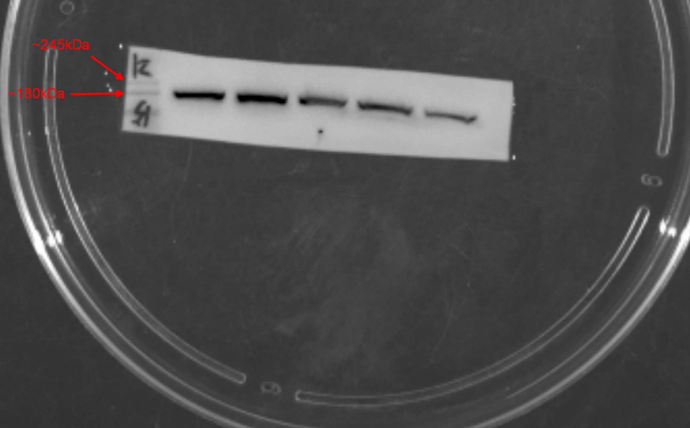

Original Figure5B-1, 5E-1, 5H-1 (β-actin)

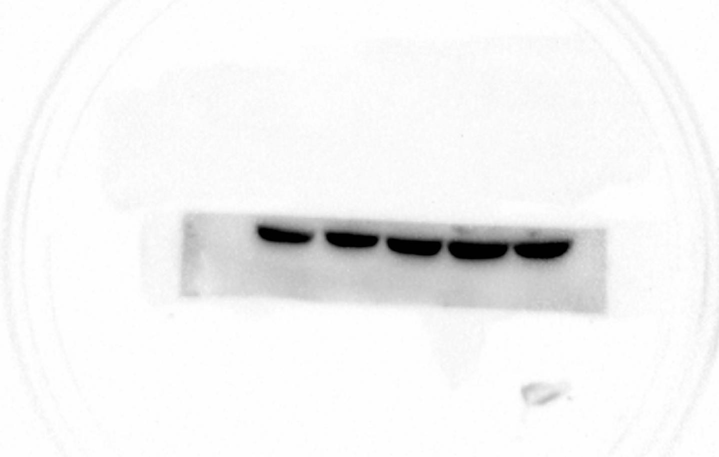

Original Figure5B-1, 5E-1, 5H-1 merged with WL-FOV (β-actin)

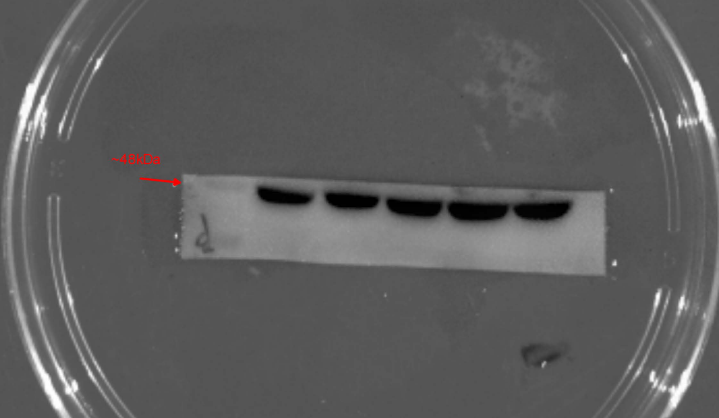

Original Figure5B-2, 5E-2, 5H-2 (β-actin)

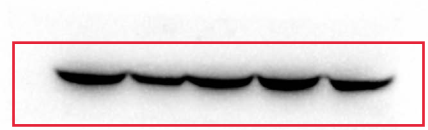

Original Figure5B-2, 5E-2, 5H-2 merged with WL-FOV (β-actin)

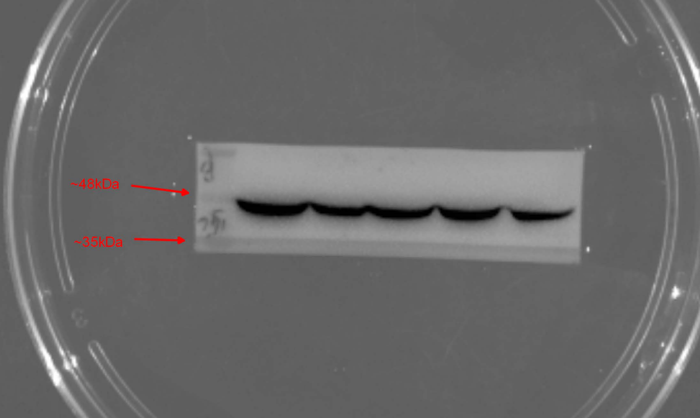

Original Figure5B-3, 5E-3, 5H-3 (β-actin)

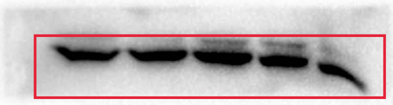

Original Figure5B-3, 5E-3, 5H-3 merged with WL-FOV (β-actin)

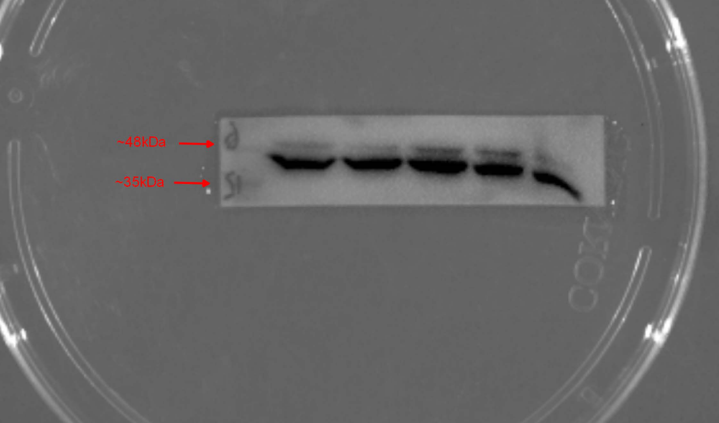

Original Figure5B-4 (Claudin-1)

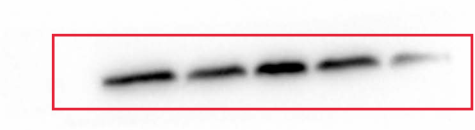

Original Figure5B-4 merged with WL-FOV (Claudin-1)

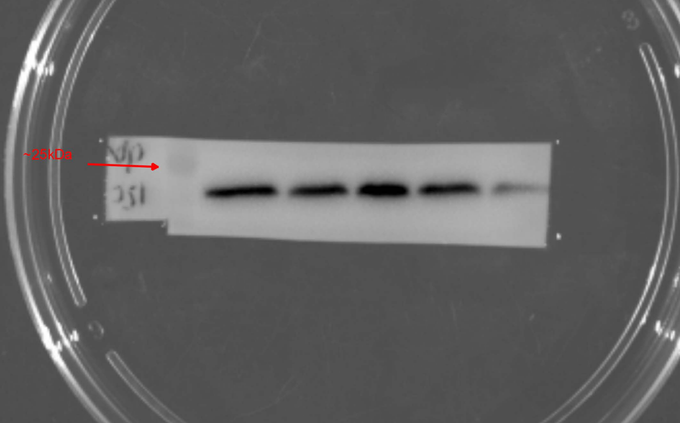

Original Figure5B-5 (Claudin-1)

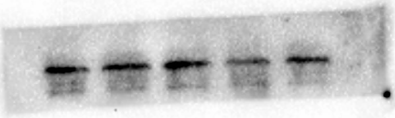

Original Figure5B-5 merged with WL-FOV (Claudin-1)

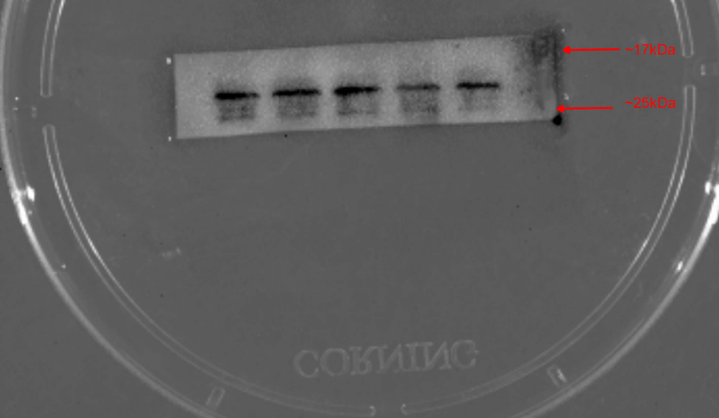

Original Figure5B-6 (Claudin-1)

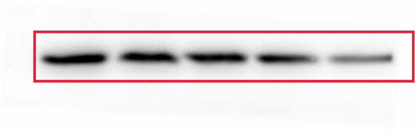

Original Figure5B-6 merged with WL-FOV (Claudin-1)

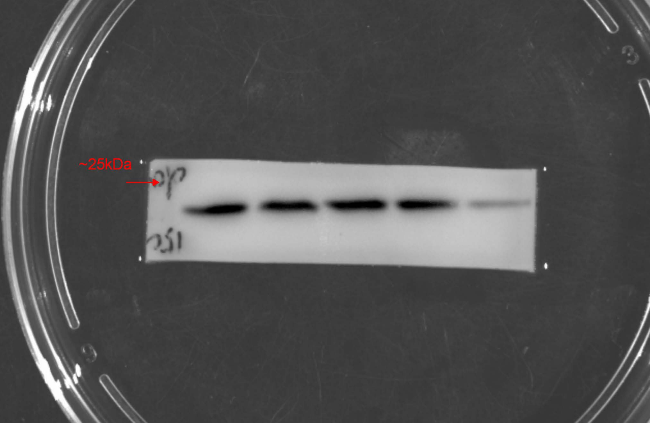

Original Figure5E-4 (Occludin)

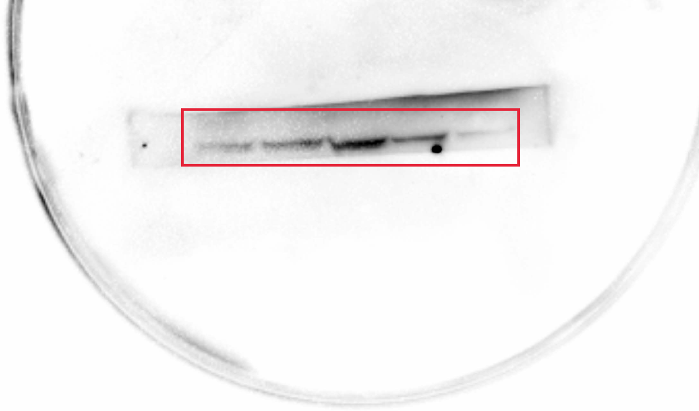

Original Figure5E-4 merged with WL-FOV (Occludin)

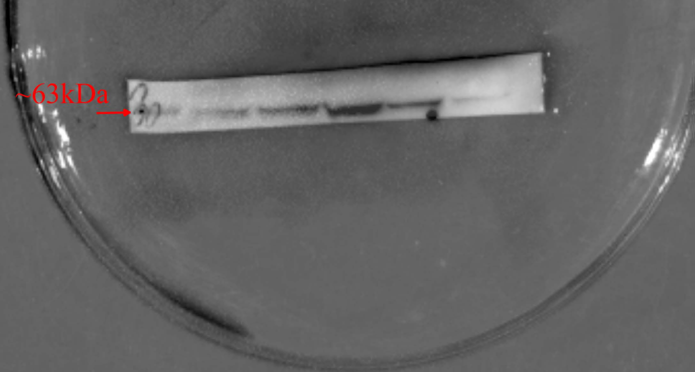

Original Figure5E-5 (Occludin)

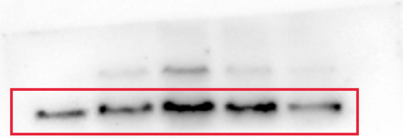

Original Figure5E-5 merged with WL-FOV (Occludin)

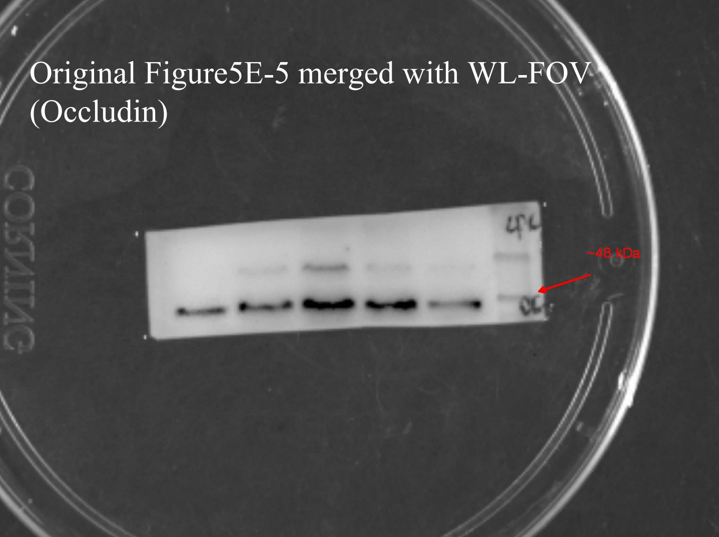

Original Figure5E-6 (Occludin)

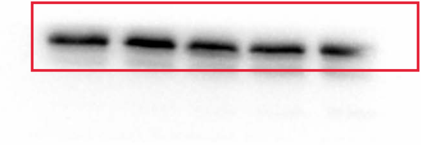

Original Figure5E-6 merged with WL-FOV (Occludin)

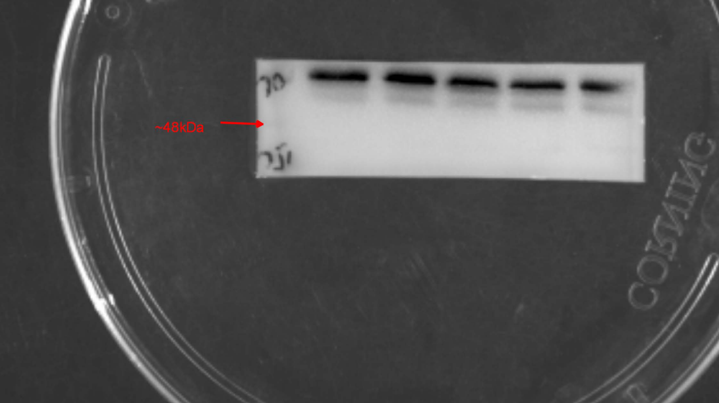

Original Figure5H-4 (ZO-1)

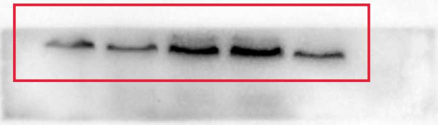

Original Figure5H-4 (ZO-1)

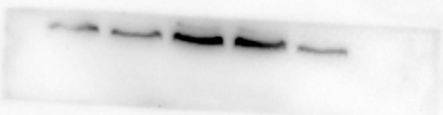

Since the WL-FOV image of this film is missing, two images with different exposure times are provided

Original Figure5H-5 (ZO-1)

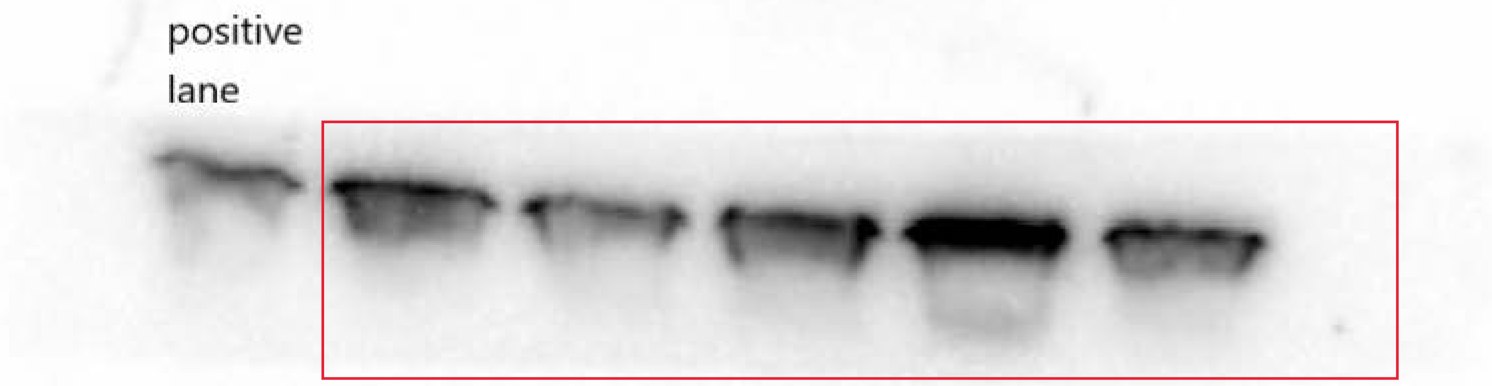

Original Figure5H-5 merged with WL-FOV (ZO-1)

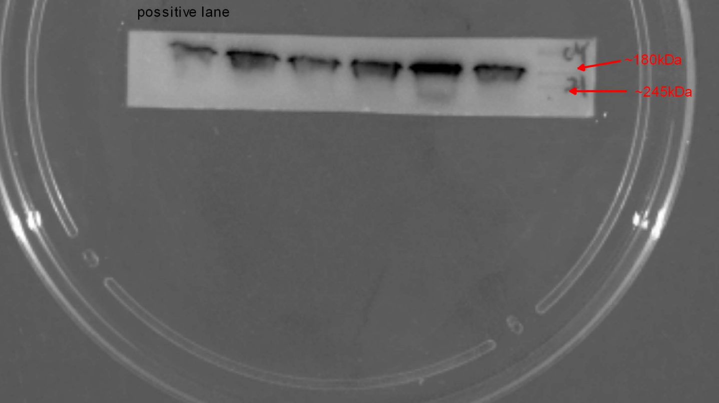

Original Figure5H-6 (ZO-1)

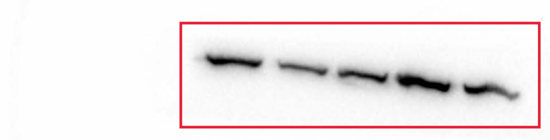

Original Figure5H-6 merged with WL-FOV (ZO-1)

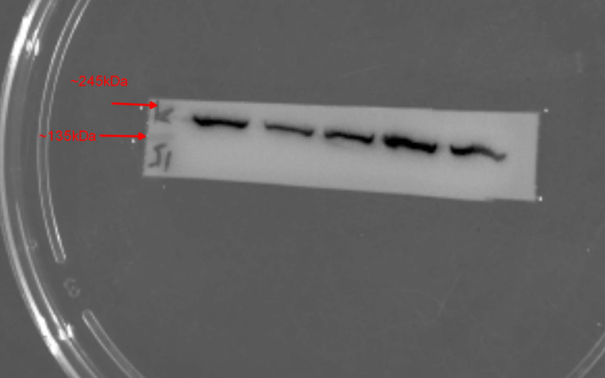

Original Figure5C-1, 5F-1, 5I-1 (β-actin)

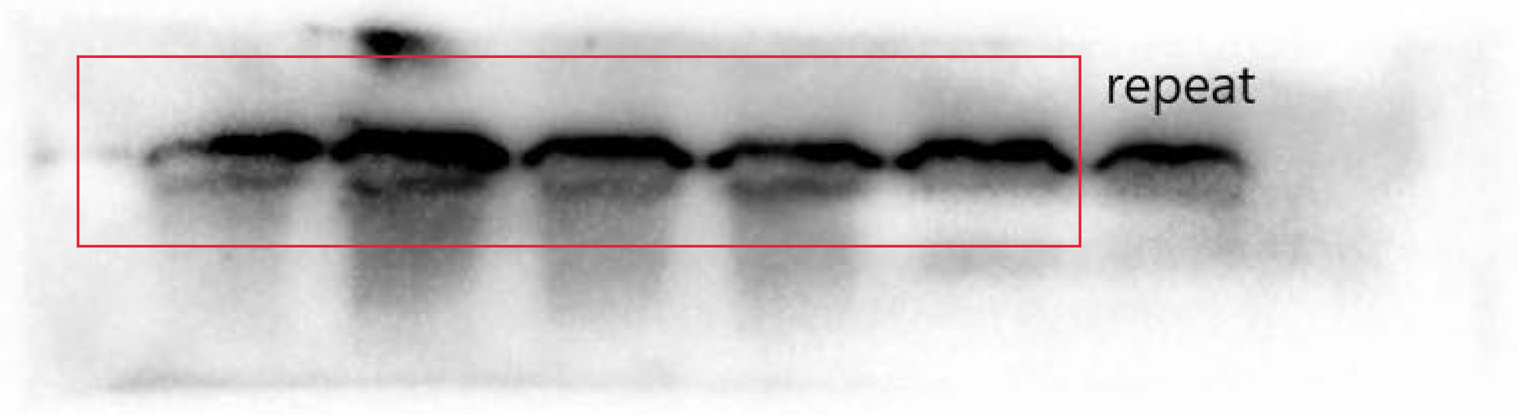

Original Figure5C-1, 5F-1, 5I-1 merged with WL-FOV(β-actin)

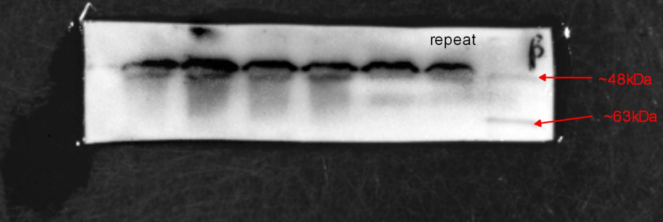

Original Figure5C-2, 5F-2, 5I-2 (β-actin)

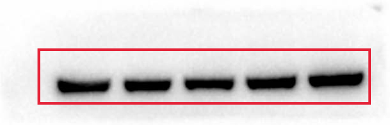

Original Figure5C-2, 5F-2, 5I-2 merged with WL-FOV(β-actin)

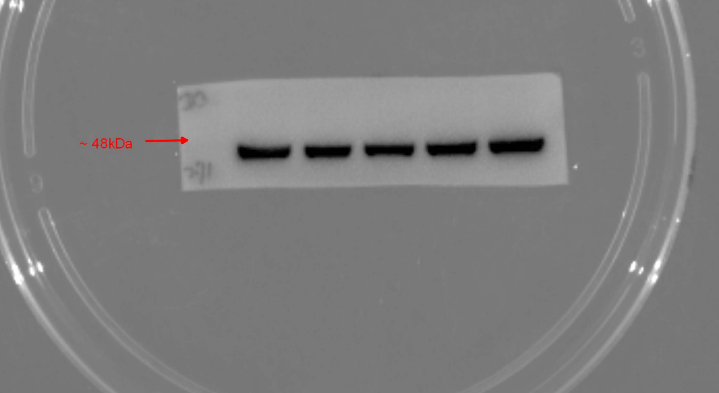

Original Figure5C-3, 5F-3, 5I-3 (β-actin)

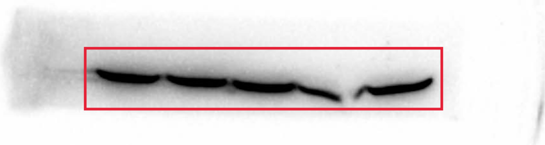

Original Figure5C-3, 5F-3, 5I-3 merged with WL-FOV(β-actin)

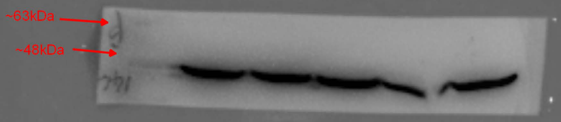

Original Figure5C-4 (Claudin-1)

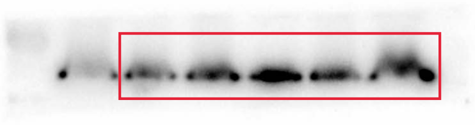

Original Figure5C-4 merged with WL-FOV (Claudin-1)

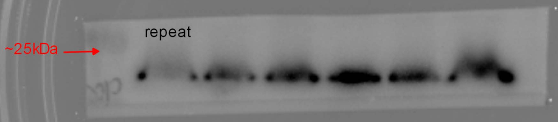

Original Figure5C-5 (Claudin-1)

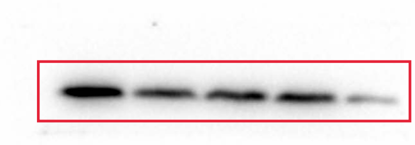

Original Figure5C-5 merged with WL-FOV (Claudin-1)

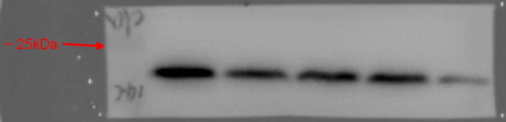

Original Figure5C-6 (Claudin-1)

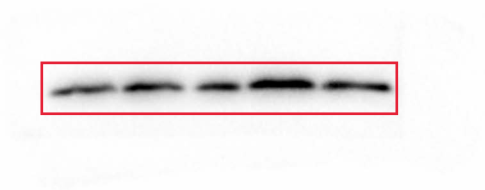

Original Figure5C-6 merged with WL-FOV (Claudin-1)

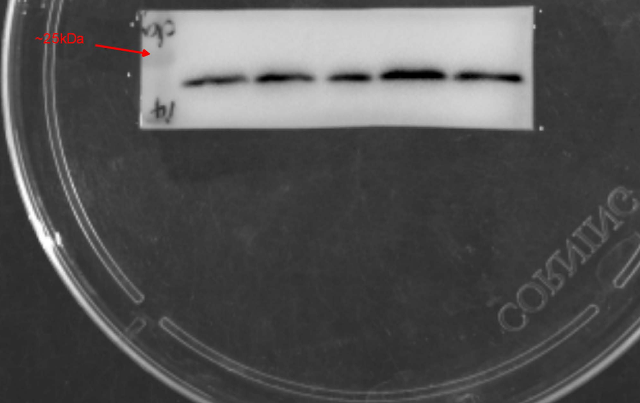

Original Figure5F-4 (Occludin)

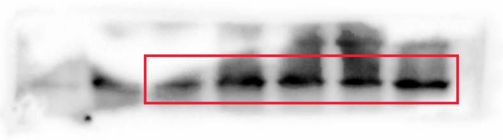

Original Figure5F-4 merged with WL-FOV (Occludin)

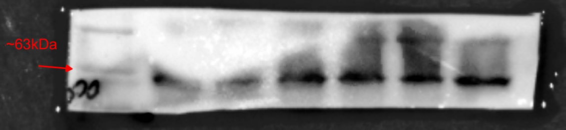

Original Figure5F-5 (Occludin)

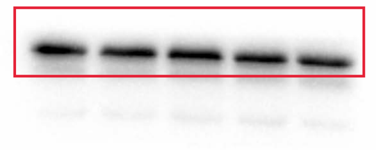

Original Figure5F-5 merged with WL-FOV (Occludin)

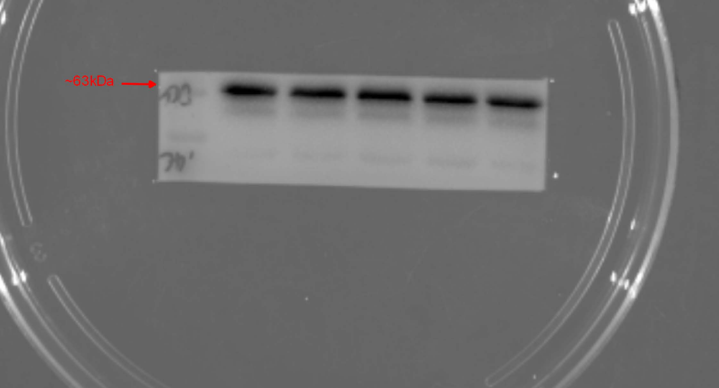

Original Figure5F-6 (Occludin)

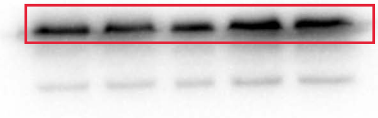

Original Figure5F-6 merged with WL-FOV (Occludin)

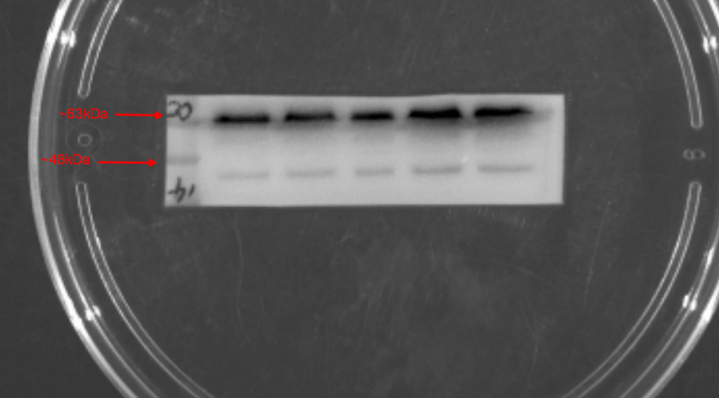

Original Figure5I-4 (ZO-1)

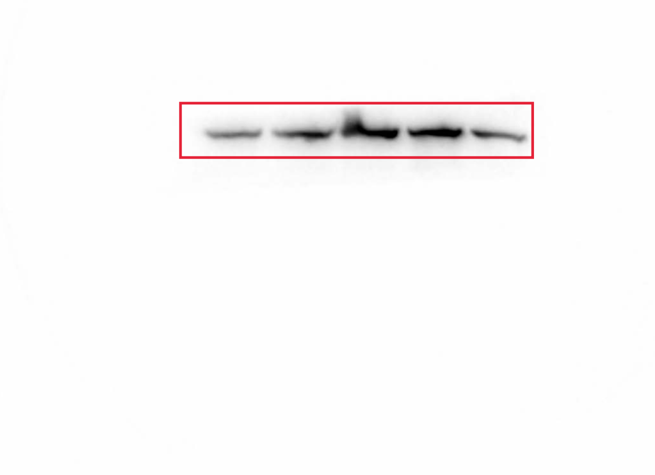

Original Figure5I-4 merged with WL-FOV (ZO-1)

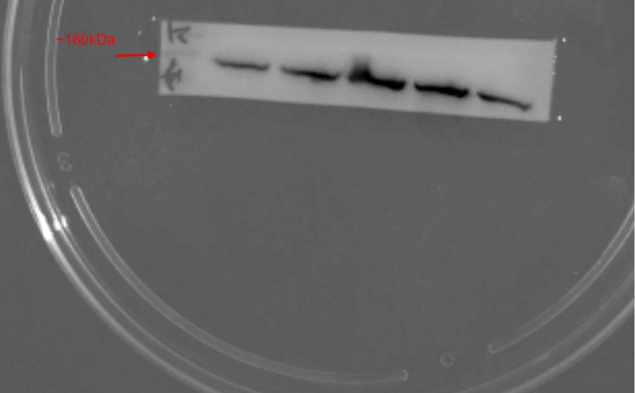

Original Figure5I-5 (ZO-1)

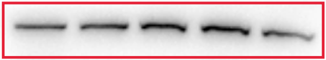

Original Figure5I-5 merged with WL-FOV (ZO-1)

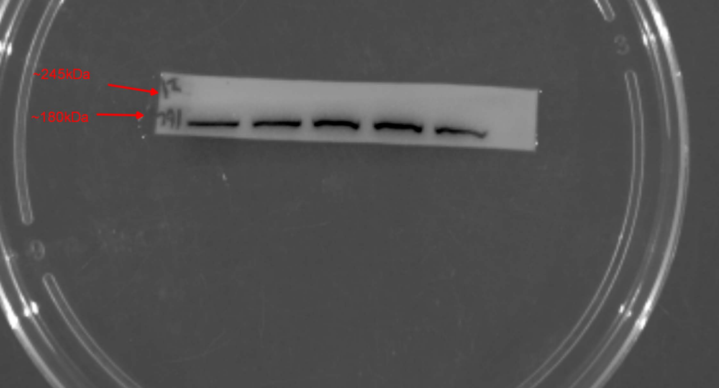

Original Figure5I-6 (ZO-1)

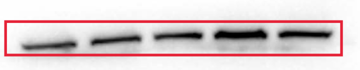

Original Figure5I-6 merged with WL-FOV (ZO-1)

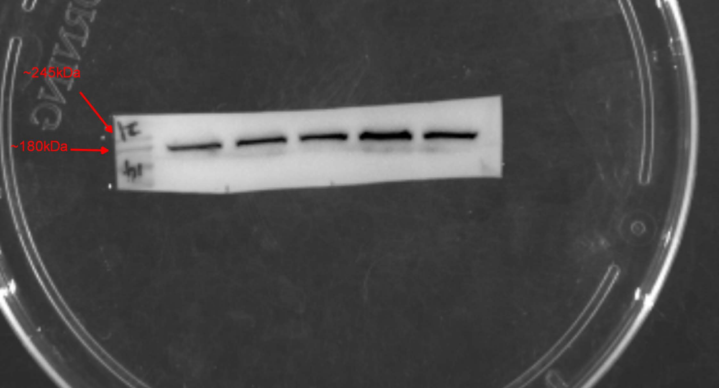

Supplement: Supplementary file 1 — Additional file 1. [file 12866_2023_2928_MOESM1_ESM.zip › original-images-figure5.pdf]
